# Construction and analysis of the chromosome-level haplotype-resolved genomes of two *Crassostrea* oyster congeners: *Crassostrea angulata* and *Crassostrea gigas*

**DOI:** 10.1093/gigascience/giad077

**Published:** 2023-10-03

**Authors:** Haigang Qi, Rihao Cong, Yanjun Wang, Li Li, Guofan Zhang

**Affiliations:** CAS and Shandong Province Key Laboratory of Experimental Marine Biology, Center for Ocean Mega-Science, Institute of Oceanology, Chinese Academy of Sciences, Qingdao 266071, China; Laboratory for Marine Biology and Biotechnology, Laoshan Laboratory, Qingdao 266237, China; National and Local Joint Engineering Key Laboratory of Ecological Mariculture, Institute of Oceanology, Chinese Academy of Sciences, Qingdao 266071, China; Shandong Technology Innovation Center of Oyster Seed Industry, Qingdao 266105, China; CAS and Shandong Province Key Laboratory of Experimental Marine Biology, Center for Ocean Mega-Science, Institute of Oceanology, Chinese Academy of Sciences, Qingdao 266071, China; Shandong Technology Innovation Center of Oyster Seed Industry, Qingdao 266105, China; Key Laboratory of Breeding Biotechnology and Sustainable Aquaculture, Institute of Hydrobiology, Chinese Academy of Sciences, Wuhan 430072, China; The Innovation of Seed Design, Chinese Academy of Sciences, Wuhan 430072, China; Marine Science Data Center, Institute of Oceanology, Chinese Academy of Sciences, Qingdao 266071, China; CAS and Shandong Province Key Laboratory of Experimental Marine Biology, Center for Ocean Mega-Science, Institute of Oceanology, Chinese Academy of Sciences, Qingdao 266071, China; Shandong Technology Innovation Center of Oyster Seed Industry, Qingdao 266105, China; Key Laboratory of Breeding Biotechnology and Sustainable Aquaculture, Institute of Hydrobiology, Chinese Academy of Sciences, Wuhan 430072, China; The Innovation of Seed Design, Chinese Academy of Sciences, Wuhan 430072, China; University of Chinese Academy of Sciences, Beijing 100049, China; CAS and Shandong Province Key Laboratory of Experimental Marine Biology, Center for Ocean Mega-Science, Institute of Oceanology, Chinese Academy of Sciences, Qingdao 266071, China; Laboratory for Marine Biology and Biotechnology, Laoshan Laboratory, Qingdao 266237, China; National and Local Joint Engineering Key Laboratory of Ecological Mariculture, Institute of Oceanology, Chinese Academy of Sciences, Qingdao 266071, China; Shandong Technology Innovation Center of Oyster Seed Industry, Qingdao 266105, China

**Keywords:** oyster, *Crassostrea angulata*, *Crassostrea gigas*, trio-binning, haplotype-resolved genome

## Abstract

**Background:**

The Portuguese oyster *Crassostrea angulata* and the Pacific oyster *C. gigas* are two major *Crassostrea* species that are naturally distributed along the Northwest Pacific coast and possess great ecological and economic value. Here, we report the construction and comparative analysis of the chromosome-level haplotype-resolved genomes of the two oyster congeners.

**Findings:**

Based on a trio-binning strategy, the PacBio high-fidelity and Illumina Hi-C reads of the offspring of the hybrid cross *C. angulata* (♂) × *C*. gigas (♀) were partitioned and independently assembled to construct two chromosome-level fully phased genomes. The assembly size (contig N50 size, BUSCO completeness) of the two genomes were 582.4 M (12.8 M, 99.1%) and 606.4 M (5.46 M, 98.9%) for *C. angulata* and *C. gigas*, respectively, ranking at the top of mollusk genomes with high contiguity and integrity. The general features of the two genomes were highly similar, and 15,475 highly conserved ortholog gene pairs shared identical gene structures and similar genomic locations. Highly similar sequences can be primarily identified in the coding regions, whereas most noncoding regions and introns of genes in the same ortholog group contain substantial small genomic and/or structural variations. Based on population resequencing analysis, a total of 2,756 species-specific single-nucleotide polymorphisms and 1,088 genes possibly under selection were identified.

**Conclusions:**

This is the first report of trio-binned fully phased chromosome-level genomes in marine invertebrates. The study provides fundamental resources for the research on mollusk genetics, comparative genomics, and molecular evolution.

## Introduction

Mollusca is the second largest phylum in the animal kingdom and contains the highest number of marine invertebrates. Oysters are filter-feeding bivalves belonging to the family Ostreidae. They are widely distributed in shallow seas and estuaries and constitute an essential component of marine ecosystems. Oysters in the genus *Crassostrea* are of special significance, as they can grow together by settling on each other's shells and forming massive reefs, which are similar to coral reefs in terms of their ecological importance. With a long history as a human food source, oysters play a considerable role in the fishery and aquaculture industries. The Pacific oyster *Crassostrea gigas* (Thunberg, 1793) (NCBI:txid29159; marinespecies.org:taxname:140,656) and Portuguese oyster *Crassostrea angulata* (Lamarck, 1819) (NCBI:txid558553; marinespecies.org:taxname:146,900) are two dominant *Crassostrea* species. They are known as cupped oysters, naturally inhabiting the Northwest Pacific coast. In China, *C. gigas* is found on the northern coast of the Yangtze Estuary, whereas *C. angulata* is found in the south of the Yangtze Estuary [1]. Although *C. angulata* was first identified and named in Portugal, early studies proved that European *C. angulata* originated from Asia [2–4] and could be considered a subspecies of *C. gigas*. In China and some recent studies, *C. angulata* was often called the Fujian oyster, and a recommendation for renaming it to *C. gigas angulata* has been proposed [1]. As our focus was not on the oyster taxonomy, in the study, we still use the words “two species” to refer to them. Their annual production has reached 4.0 million tons since 2004 [5], and they have been the oyster species with the highest consumption and trade volume.

*C. gigas* was considered a model organism in Lophotrochozoa [5] and among the first batch of mollusk species with an accessible whole-genome assembly [6]. As a first release, the *C. gigas* genome version “v9” (GenBank Acc No: GCA_000,297,895.1) has brought convenience to oyster basic research. However, due to technical limitations, the “v9” assembly was highly fragmented and contained some assembling or annotation errors [7, 8], despite the use of an oyster derived from four generations of full-sibling mating and a fosmid-pooling hierarchical assembly strategy. Two chromosome-level *C. gigas* genomes have recently been published [9, 10], which have improved the assembly quality and further expanded the genomic resources for the research community. Several studies have been conducted to explore the differentiation of the two species [11–13], but the *C. angulata* genome is yet to be available, and a full comparison with *C. gigas* at the whole-genome sequence level is lacking. This, to some degree, limits our understanding of the genomics and evolution of *Crassostrea* oysters.

Before 2015, only a few mollusk genomes were published. With the rapid development of sequencing and scaffolding technologies, it is feasible to complete chromosome-level genome assembly at a relatively low cost for nonmodel organisms. The number of mollusk genomes started to explode in 2017 [14]; in the past 1 to 3 years, chromosome-level genomes of Ostreidae oysters have been massively released, including the Pacific oyster *C. gigas* [9, 10], the Jinjiang oyster *Crassostrea ariakensis* [15, 16], the Hong Kong oyster *Crassostrea hongkongensis* [17], and the European flat oyster, *Ostrea edulis* [18, 19]. These genomes have covered most of the oyster species that are of great ecological and economic value throughout the world.

A trend for genome assembling is to construct the haplotype-resolved (phased) sequences, which are more favorable for variation discovery and genetic dissection of complex traits than the traditional “squashed” or “mosaic” genomes [20, 21]. Phasing in highly divergent regions can be achieved by utilizing single-nucleotide polymorphism (SNP) allele linkage information through a variety of programs [22]. However, building a fully phased genome is far more challenging. The complete high-quality haplotype-resolved genomes have been accomplished in several species in the past few years [20, 21, 23, 24]. This is largely ascribed to the advent of technology producing highly accurate long DNA sequences represented by the PacBio high-fidelity (HiFi) sequencing method, in that the once-widely used long reads are noisy with 10% to 15% error rates. Additionally, phasing was usually lost after corrections [25]. The development of assembly methods or algorithms, such as trio-binning [26], DipAsm [27], and Hifiasm [28], has facilitated the production of haplotype-resolved genomes.

In the present study, we adopted a trio-binning strategy to build two chromosome-level haplotype-resolved genomes for two *Crassostrea* oyster congeners—*C. angulata* and *C. gigas*—and conducted a comparative genomic analysis. To the best of our knowledge, this is the first report of a fully phased mollusk genome and may further benefit research on molecular ecology, evolution, and genetics in mollusks.

## Materials and Methods

### Sample collection and sequencing

One hybrid full-sib family was produced by mating a male *C. angulata* (hereinafter referred to as “AN”) oyster from Xiamen, China, with a female *C. gigas* (“GI”) oyster from Qingdao, China. The two parents and a one-year-old offspring (“CH1”) were sampled and used for sequencing. Genomic DNA was extracted from the mantle tissues using the standard phenol–chloroform method. Library preparation, quality control, and sequencing were performed according to standard protocols. Short paired-end DNA reads from a whole genome shotgun (WGS) library with an insert size of 300 bp were produced for AN, GI, and CH1 using the Illumina NovaSeq 6000 system (RRID:SCR_016387). Short paired-end DNA reads of CH1 were produced from a high-throughput chromosome conformation capture (Hi-C) library with an insert size of 500 bp using the Illumina NovaSeq 6000 system. Long DNA reads from a library with an insert size of 15 to 20 kbp were generated using the PacBio Smart Sequel II platform (RRID:SCR_017990). The highly accurate consensus sequence (HiFi) reads were obtained using ccs software (RRID:SCR_021174) version 6.0.0. High-quality short paired-end DNA reads were obtained using fastp software (RRID:SCR_016962) version 0.21.0 [29] with the parameters of “-q 20 -u 30 -n 0 -e 20.”

### Genome survey and reads partition

Basic genome features, including genome size, heterozygosity rate, and repeat content, were estimated by *k*-mer–based methods using GenomeScope software (RRID:SCR_017014) version 2.0 [30]. The partitioning of CH1 reads by AN- and GI-unique *k*-mers was conducted using K-Mer Counter software version 3.1.1 [31]. Briefly, the *k*-mer database of *k*-mer size of L for AN (KL_A_) and GI (KL_B_) was made at L = 25, 50, 75, and 100 bp by the “kmc” command with parameters of “-t 20 -ci 1 -cs 1000.” The unique *k*-mer database (KLU) was obtained via set difference operation (i.e., AN unique *k*-mer database KLU_A_ = KL_A_− KL_B_, GI unique *k*-mer database KLU_B_ = KL_B_ − KL_A_, by “kmc_tools kmers_subtract” command with parameters of “-ci 30 -cx 300”). Then the distribution of KLU_A_ and KLU_B_  *k*-mers was counted for each CH1 read by the “kmc_tools intersect” command. For PacBio HiFi reads, at least two KLU supports were required to group a read. On the other hand, only one KLU support was required for short sequencing reads. Reads without any KLU *k*-mers were considered common reads. Finally, common reads and KLU_A_-containing reads were grouped as AN reads; whereas common reads and KLU_B_-containing reads were grouped as GI reads.

### Assembling and assessment

The partitioned PacBio HiFi reads of AN and GI were separately assembled using Hifiasm (RRID:SCR_021069) version 0.16.1-r375 with default parameters [28]. The assembly errors of the AN and GI contigs were examined and corrected using Inspector version 1.0.1. Possible contaminants in contigs were detected using the contaminant screening system on the NCBI genome submission website and then were removed or fixed manually. Next, the Hi-C DNA reads of AN and GI were mapped to the AN and GI cleaned contigs, respectively, using BWA (RRID:SCR_010910) version 0.7.17-r1188 [32], and the Hi-C contact matrix was constructed using Juicer (RRID:SCR_017226) version 1.5 [33]. Finally, 3D-DNA version 180,922 [34] was used to further detect and correct the assembly errors, infer the order and orientation of each contig, and link them to chromosome-level scaffolds.

The quality of the final assembly was evaluated as follows. (i) Metazoan BUSCO genes. Quality assessment was conducted using BUSCO software (RRID:SCR_015008) version 5.2.2 [35] with default parameters except a stringent e-value of “1e-5” by searching the genome against 954 metazoan single-copy orthologs from metazoa_odb10 [36]. (ii) Short DNA reads mapping. Short WGS DNA reads of AN and GI were mapped to the two genomes using BWA. In addition, short DNA reads of 20 *C. gigas* individuals from Qingdao and 20 *C. angulata* individuals from Ningde (Supplementary File 1) were mapped to the two genomes. The percentage of mapped reads and unique mapped reads, breadth coverage at single base depth ≥1, and breadth coverage at a single base depth ≥4 were calculated based on the BAM file using Samtools software (RRID:SCR_005227) version 1.9 [37] to roughly assess the representative of the genome. (iii) Transcriptome mapping. A total of 18 *C. angulata* transcriptomes and 18 *C. gigas* transcriptomes in NCBI PRJNA516773 were mapped to the two genomes using the Hisat2 program (RRID:SCR_015530) version 2.1.0 [38]. For a comparison, 18 transcriptomes from another *Crassostrea* oyster, *C. ariakensis* in NCBI PRJNA513213, were mapped to the two genomes. The basic mapping statistics were summarized to assess the performance of the genome as a reference for RNA sequencing (RNA-seq) analysis.

### Genome annotation

*De novo* and homology-based transposable elements (TEs) or interspersed repeats prediction were conducted using RepeatModeler (RRID:SCR_015027) version 2.0.3 [39] and RepeatMasker (RRID:SCR_012954) version 4.1.2 [40], respectively. Tandem Repeats Finder (TRF) (RRID:SCR_022193) version 4.09 [41] was used to detect simple or tandem repeats. The TE-masked genome was used for gene model prediction using homolog-based and RNA-seq–based approaches as described in our previous study [6, 10].

Functional annotation of the predicted genes was conducted by means of 5 widely used datasets. NCBI “nonredundant” (NR), Swiss-Prot, and KEGG annotations were retrieved by aligning the proteins to the corresponding database using BlastP [42] with an E-value threshold of 1e-5; the best hit was retained. Protein domain annotation was executed by searching the InterPro database (RRID:SCR_006695) using InterProScan (RRID:SCR_005829) version 5.34–73.0. GO annotations for each gene were obtained by mapping the InterPro entries to GO terms according to the “interpro2go” file.

### Comparative genomics and evolutionary analysis

To assess the global similarity of the *C. angulata* and the *C. gigas* genomes, a direct DNA sequence comparison between the 10 largest scaffolds (pseudo-chromosome sequences 1–10) of AN and GI genome assembly was conducted using minimap2 version 2.15-r905 with the parameters of “-t 10 -c -N 2 -Y –eqx -x asm20” [43]. The corresponding fragments whose aligned-region size between two homologous pseudo-chromosome scaffolds were greater than 1,000 bp were retained and their sequence similarity was measured by the BLAST identity (gap-uncompressed method, defined as the proportion of identical bases in the full length of the alignments, and the gap-compressed identity [gap-compressed method, where consecutive gaps are counted as 1 gap]) [44]. The sequence divergence rate was calculated by subtracting the sequence identity.

The *C. angulata* and *C. gigas* gene sets, together with gene sets of 13 other mollusk species (11 bivalves and 2 gastropods) and 1 annelid species from a public database (Supplementary Table S1), were collected and ortholog groups were constructed using OrthoFinder (RRID:SCR_017118) version 2.3.12 [45]. For *C. angulata* and *C. gigas* coding gene comparison, ortholog gene pairs were extracted from the *C. angulata*–*C. gigas* orthogroups. All the possible gene pairs in orthogroups were compared, and genes with the same number of coding sequences (CDS) were considered to have the same gene model structures. The predicted peptide sequences, coding sequences, intron sequences, and upstream and downstream sequences of the orthologous gene pairs were aligned using Muscle (RRID:SCR_011812) version 3.8.1551 [46], and the sequence identities were measured by BLAST identity as mentioned above.

To deduce the divergence time of *C. angulata* and *C. gigas*, the single-copy orthologous genes shared by the 16 genomes (Supplementary Table S1) were aligned using Muscle version 3.8.1551 [46] and then concatenated to construct a maximum likelihood phylogenetic tree using IQ-TREE (RRID:SCR_017254) version 2.2.0 with the parameters of “-m MFP -T 40 -B 10,000 –alrt 10,000 -bnni” [47]. The divergence time of species was estimated using the MCMCTree program in PAML package (RRID:SCR_014932) version 4.7a [48] (the control parameters are listed in Supplementary File 2). Reference divergence time values (*C. gigas–C. virginica*: 63–83 million years ago (MYA) [49, 50]; *Elysia chlorotica–Pomacea canaliculata*: 343–478 MYA) retrieved from the TimeTree database (RRID:SCR_021162) [51] were used to calibrate divergence times on the phylogenetic tree.

To infer the selective pressure of the coding genes, the protein sequences of the ortholog gene pairs between *C. angulata* and *C. gigas* were aligned using Muscle software and based on the amino acid alignments from which the nucleotide codon alignments were retrieved using the PAL2NAL program [52]. The nonsynonymous substitution rate (Ka or d_N_), synonymous substitution rate (Ks or d_S_), and the ratio of nonsynonymous to synonymous substitution rates (Ka/Ks or d_N_/d_S_) were calculated using KaKs_Calculator 2.0 with the “NG” methods [53]. The Ka/Ks values were considered statistically significant with a *P* < 0.05 evaluated with a Fisher exact test.

For protein domain abundance analysis of the 16 genomes, the protein sequences of the predicted coding genes of each genome were aligned to the conserved domain or family profiles in the PFAM database [54] using HMMER (RRID:SCR_005305) version 3.3.2 [55] with a E-value cutoff of 1e-5. Proteins sharing the same domains were clustered into a single gene set and the gene numbers (GNs) in each species were determined. We defined the species-expanded protein domains using the following criteria: (i) the species with the largest GN, (ii) the ratio of maximum GN to second maximum GN was greater than 1.2, (iii) the difference between the maximum GN and the second maximum GN was above 3, and (iv) the ratio of the maximum GN to the average GN of the other species was above 1.5.

A total of 47 wild *C. angulata* oysters collected in Xiamen, China, in October 2022 were resequenced with a raw base production of 15 to 20 G in the study. Together with 22 *C. angulata* samples from Ningde and 33, 20, and 33 *C. gigas* oysters from Qingdao, Yantai, and Jinzhou, respectively, produced in our previous study [56], a collection of 69 *C. angulata* and 86 *C. gigas* oysters (Supplementary File 1) was used for the resequencing analysis. Briefly, the high-quality resequencing reads were extracted for each individual using fastp software, then mapped to the *C. gigas* genome using the BWA program. Subsequently, the bam files were sorted, duplications were removed and indexed using the samtools program, and SNPs were called using GATK version 4.1.9.0 [57] with parameters of “QD < 2 || FS > 60 || MQ < 40 || MQRankSum < -8 || ReadPosRankSum < -8.” A phylogenetic tree was constructed using FastME version 2.0 [58], and the population structure was inferred using ADMIXTURE (RRID:SCR_001263) version 1.3.0 [59]. The nucleotide diversity (θ_π_) and wright's fixation index (*F_ST_*) were estimated using VCFtools (RRID:SCR_001235) version 0.1.16 [60]. Linkage disequilibrium decay was analyzed using PopLDdecay (RRID:SCR_022509) version 3.42 [61]. An SNP was considered species enriched if the absolute value of the allele frequency difference between the two populations was greater than 0.75 and was considered species specific if the value was greater than 0.95.

## Results

### Genome sequencing

A total of 383 G DNA sequencing bases were produced, including 88 G (roughly 147×) bases from 5.49 M highly accurate long DNA reads (HiFi reads) produced by PacBio circular consensus sequencing technology and 295 G Illumina short DNA reads (Table 1). The average length of HiFi reads reached 16 kbp, the average phred-scaled base quality (BQ) was 29.9, and the Q20 and Q30 base percentages were comparable to those of Illumina short DNA reads. During consensus sequencing calling, the average phred-scaled read quality (RQ) and the pass number (PN) were highly positively correlated (Pearson correlation = 0.997, *P* < 1.0e-10); when the PN thresholds were 3, 4, and 5, the mean RQs were 29.9, 30.1, and 30.7, respectively.

**Table 1:** Sequencing data summary

	AN	GI	CH1		
Species	*C. angulata* (♂)	*C. gigas* (♀)	*C. angulata* × *C. gigas* offspring		
Read Type	Short DNA	Short DNA	Short DNA	Hi-C DNA	PacBio HiFi
Raw Seq (M)	407.80	430.63	470.65	804.98	97.94
Raw Bases (G)	61.17	64.59	70.60	120.75	1404.32
Filtered Seq (M)	366.74	383.34	446.80	770.32	5.49
Filtered Bases (G)	55.01	57.50	67.02	115.55	88.01
Filtered Q20 (%)	97.86	98.01	97.66	98.28	98.13
Filtered Q30 (%)	93.40	93.83	93.11	94.39	95.71
Average size (bp)	150	150	150	150	16,032
Coverage (×)	92	96	112	193	147

### Genome survey and reads partition

**Figure 1:**
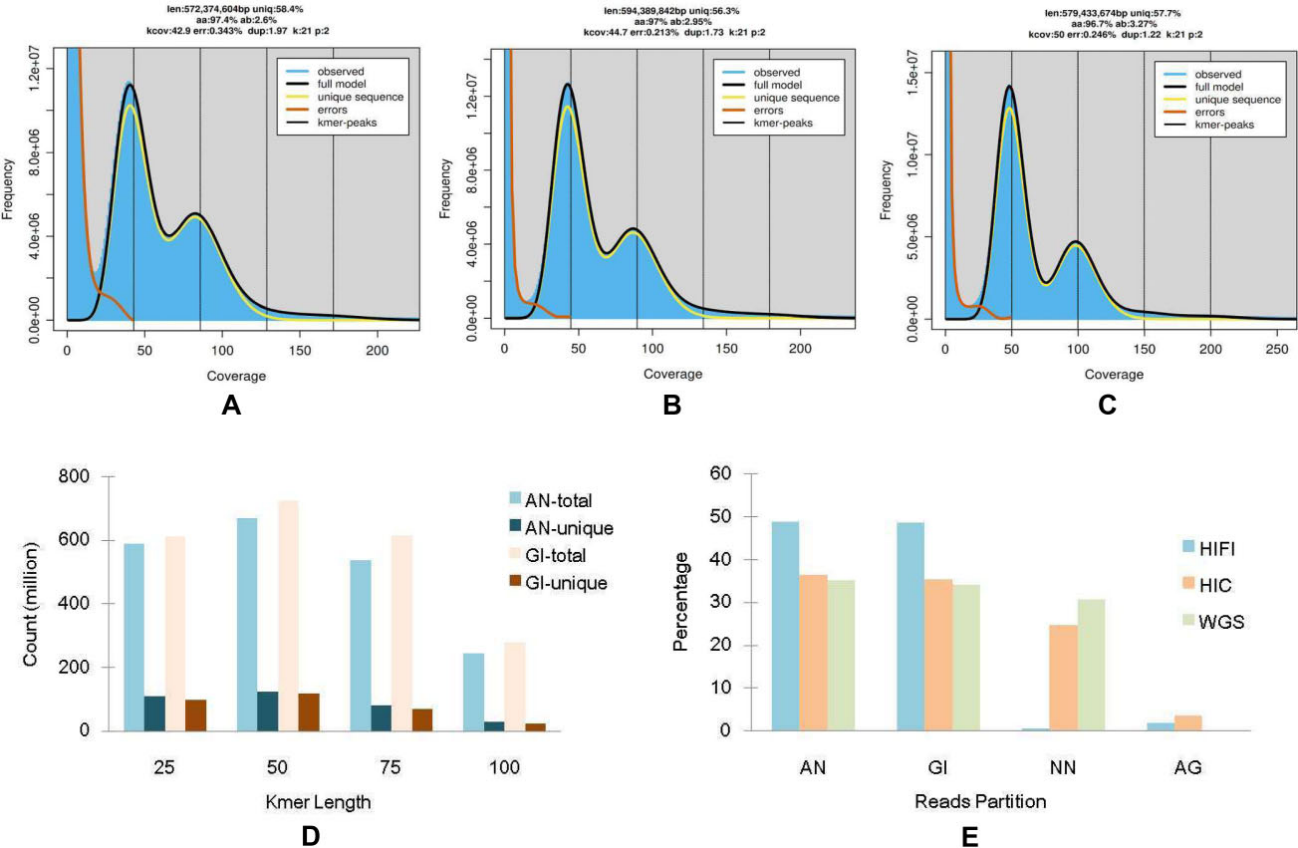
Genome feature survey and trio sequencing reads partition based on *k*-mer analysis. (A–C) GenomeScope analysis for male *C. angulata* (A), female *C. gigas* (B), and the hybrid offspring (C), respectively. x-axis: the sequencing reads coverage. y-axis: the 21-mer frequency. (D) The distributions of 25-, 50-, 75-, and 100-mers in the AN (*C. angulata*) and GI (*C. gigas*) short sequencing reads. x-axis: *k*-mer length. y-axis: *k*-mer count. (E) The offspring reads partition by unique *k*-mers. x-axis: reads partition types. y-axis: the percentage of the reads. AN: reads that only contain *C. angulata* unique *k*-mers. GI: reads that only contain *C. gigas* unique *k*-mers. NN: reads that do not contain *C. angulata* or *C. gigas* unique kmers. AG: reads that contain *C. angulata* and *C. gigas* unique *k*-mers.

The estimated genome size, heterozygosity rate, and repeat content of AN, GI, and CH1 were 572.3 M (2.6%, 41.6%), 594.4 M (2.9%, 43.7%), and 579.4 M (3.3%, 42.3%), respectively (Fig. 1A–C). As expected, the genome size and repeat content of CH1 were both approximately the average of those of the two parents, whereas the CH1 heterozygosity rate was significantly higher than that of AN and GI.

A large number of unique *k*-mers were found in the AN and GI WGS DNA reads (Fig. 1D). For AN, at *k*-mer sizes of 25, 50, 75, and 100 bp, there were approximately 590, 670, 538, and 246 M *k*-mers, respectively, and the corresponding unique *k*-mer numbers were 110, 124, 79, and 29 M, respectively. For GI, there were approximately 614, 725, 616, and 279 M *k*-mers, and the unique *k*-mer numbers were 99, 118, 69, and 23 M, respectively. Although the total number of GI *k*-mers was slightly higher than that of AN, the number of unique *k*-mers of GI was lower than that of AN. In AN and GI, the unique *k*-mers accounted for 11.7% to 18.6% and 8.2% to 16.1%, respectively, of the total *k*-mers.

All types of CH1 sequencing reads were grouped using AN- and GI-unique *k*-mers to constitute AN- and GI-originated read sets (Fig. 1E). More than 97% of the HiFi reads can be effectively partitioned, including 48.9% of AN reads, 48.7% of GI reads, and 0.6% of common reads. For the Hi-C short DNA reads, the AN, GI, and common reads accounted for 36%, 35%, and 25%, respectively, of the total reads. Similarly, for the WGS short DNA reads, the AN, GI, and common reads accounted for 35%, 34%, and 31%, respectively, of the total reads.

### Genome assembly and assessment

**Figure 2:**
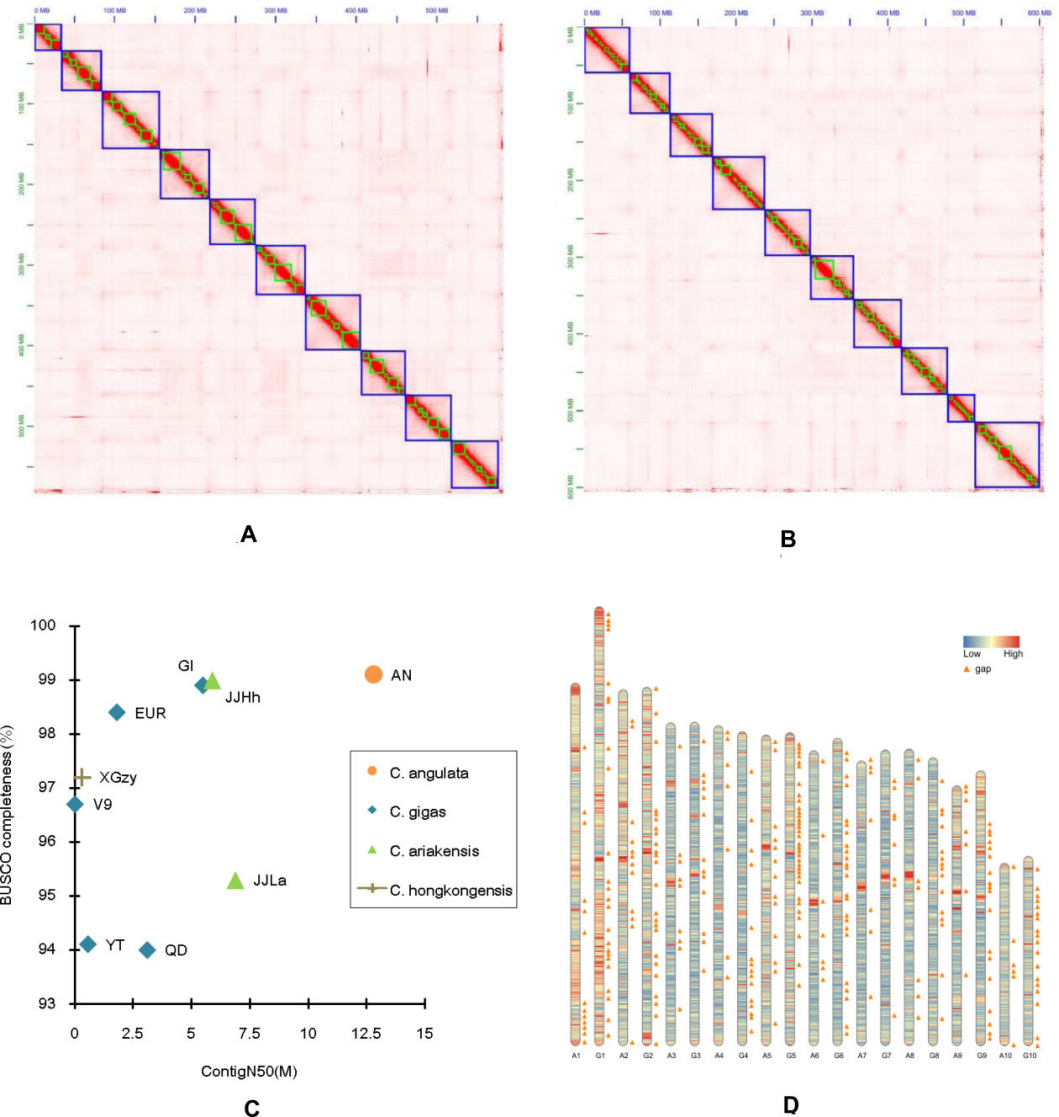
Genome assembling and assessment. (A, B) The heatmap for the Hi-C contact matrix of *C. angulata* (A) and *C. gigas* (B), respectively. The x-axis and y-axis denote the assembly size. The color scale in the heatmap corresponds to the normalized reads counts representing the 3-dimensional proximity of pairs of contigs in the genome. A bright diagonal is the dominant visual feature indicating that most of the contigs were well placed. (C) BUSCO evaluation and contig N50 of several published *Crassostrea* genomes. x-axis: the contig N50 size. y-axis: the BUSCO completeness. AN: *C. angulata* genome produced in the study. GI: *C. gigas* genome produced in the study. QD: *C. gigas* genome (GCA_011,032,805.1). EUR: *C. gigas* genome (GCA_902,806,645.1). YT: *C. gigas* genome (GCA_005,518,195.2). V9: *C. gigas* genome (GCA_000,297,895.1). JJLa: *C. ariakensis* genome (GCA_020,567,875.1). JJHh: *C. ariakensis* genome (GCA_020,458,035.1). XGZy: *C. hongkongensis* genome (PRJNA592306). (D) The schematic diagram of the 10 pairs of pseudo-chromosome sequences of the two genomes. A1–A10: The pseudo-chromosomes (10 largest scaffolds: canscf1–canscf10) in *C. angulata* genome. G1–G10: The pseudo-chromosomes (10 largest scaffolds: cgiscf1–cgiscf10) in *C. gigas* genome. Low (blue) to high (red) gradual colors: repeats content in 50-kbp sliding windows. Orange triangle: the gaps between contigs.

Portioned HiFi reads of CH1 were used to independently construct contigs with high contiguity for the two parents (AN and GI) to generate two fully haplotype-resolved or phased genome assemblies. Using the portioned Hi-C reads of CH1, the 3-dimensional proximities of the contig pairs in each phased genome were deduced from the Hi-C contact matrix, and most contigs were well placed in the scaffolding process (Fig. 2A, B). The assembly size and contig N50 of the AN genome were 582.3 M and 12.7 M, respectively, and those of the GI genome were 606.3 M and 5.5 M, respectively (Table 2; Fig. 2C).

**Table 2:** Assembly statistics of the two haplotype-resolved genomes.

	*C. angulata*		*C. gigas*	
	Contig	Scaffold	Contig	scaffold
Sequence Number	166	75	293	88
Assembly Size (M)	582.28	606.27		
Longest SeqLen (M)	22.47	70.14	25.30	84.89
Shortest SeqLen (K)	13.94	14.50	16.33	19.02
Average SeqLen (M)	3.51	7.76	2.07	6.89
N50 (M)	12.78	60.09	5.46	60.54
L50	18	5	35	5
N95 (M)	1.63	35.28	0.83	36.64
L95	67	10	139	10

The scaffold L95 of the two genomes was equal to 10, which is the expected haploid number of the *C. gigas* genome. This suggests that in each genome, the 10 pseudo-chromosome sequences consisting of the 10 longest scaffolds may well represent the overwhelming majority of the whole-genome contents. Similar to the *k*-mer-based genome size estimation, the assembly size of AN was approximately 24 M smaller than that of GI. On the other hand, with the exception of pseudo-chromosome 1, the size differences of the remaining corresponding pseudo-chromosome sequences of the 2 genomes were much smaller (Fig. 2D).

BUSCO assessment using 954 metazoan single-copy orthologs revealed that the proportion of complete (C), complete and single-copy (S), completely duplicated (D), fragmented (F), and missing (M) genes of the *C. angulata* genome and the *C. gigas* genome was [C:99.1%; S:98.3%; D:0.8%; F:0.6%; M:0.3%] and [C:98.9%; S:98.0%; D:0.9%; F:0.5%; M:0.6%], respectively, implying the improved assembly quality in comparison with the several previously published *Crassostrea* oyster genomes (Fig. 2C; Supplementary File 3).

Using *C. angulata* genome as a reference, the overall mapping rates of WGS short reads of AN, CH1, and GI were 98.36%, 98.21%, and 97.25%, respectively, showing a very slight gradual decreasing trend. Using *C. gigas* genome as a reference, the overall mapping rates of WGS short reads of GI, CH1, and AN were 98.54%, 98.30%, and 97.18%, respectively, showing the same trend observed above (Supplementary Table S2). At the population level, the average mapping rates of WGS short reads of 20 *C. angulata* and 20 *C. gigas* oysters using *C. angulata* as the reference were 94.16% ± 0.19% and 93.47% ± 0.45%, respectively. The average mapping rates were 93.67% ± 0.21% and 94.00% ± 0.41% when using *C. gigas* as the reference.

The mean mapping rates when mapping the transcriptome reads of *C. angulata* and *C. gigas* to the two genomes were between 73% and 77%. However, 3% to 4% differences were observed when mapping to their own genome in comparison with mapping to another genome. A similar trend was observed for unique mapping rates. In contrast, the mapping rate of mapping the transcriptome reads of the distantly related oyster, *C. ariakensis*, to the two genomes was both less than 24%, and the mapping rate of mapping the *C. angulata* and *C. gigas* transcriptome reads to *C. ariakensis* genome was both less than 18% (Supplementary Table S2).

### Repeat sequences and gene annotation

By combining repeat detection using *de novo* and homology-based methods, a total of 279.7 M of repetitive sequences were identified in the *C. angulata* genome, accounting for 48.0% of the genome. The repeat contents of the two genomes were nearly identical at the whole-genome level and between the 10 pairs of pseudo-chromosome sequences (Supplementary Table S3). In both genomes, interspersed repeats dominated and the tandem repeat percentages were less than 5%. The overall repeat content was at a medium level in the animal kingdom and was comparable to that of other *Crassostrea* genomes.

A total of 28,211 and 28,441 coding genes were predicted in the two genomes, and more than 21,584 (76.5%) and 21,740 (76.4) coding genes could be annotated using at least two types of publicly protein datasets (Table 3). The two gene sets were highly similar in terms of gene/CDS number, gene/CDS length and percentage, number of genes with different exon numbers, and number of genes with different annotations. Most of the genes (79%–80%) contained 2 to 20 exons, with single-exon genes accounting for approximately 14% and less than 7% of the genes containing more than 20 exons.

**Table 3:** Gene prediction and annotation summary of the two genomes

	*C. angulata*	*C. gigas*
Gene No.	28,211	28,441
Total CDS length (M)	42.91 (7.37%)	43.01 (7.10%)
Mean CDS length	1,521	1,512
Total gene length (M)	209.01 (35.90%)	206.48 M (34.06%)
Mean gene length	7,409	7,259
CDS 1	3,852 (13.65%)	3,963 (13.93%)
CDS 2–10	17,940 (63.59%)	18,094 (63.62%)
CDS 11–20	4,522 (16.03%)	4,522 (15.90%)
CDS >20	1,897 (6.72%)	1,862 (6.54%)
NR	27,544 (97.64%)	27,696 (97.38%)
Swiss-Prot	14,824 (52.55%)	14,763 (51.91%)
KEGG	10,210 (36.19%)	10,193 (35.84%)
InterPro	20,892 (74.06%)	20,978 (73.76%)
GO	13,884 (49.22%)	13,884 (48.82%)
No. of ≥1 annotations	27,589 (97.80%)	27,755 (97.59%)
No. of ≥2 annotations	21,584 (76.51%)	21,740 (76.44%)

### Comparative genomics and evolutionary analysis

**Figure 3:**
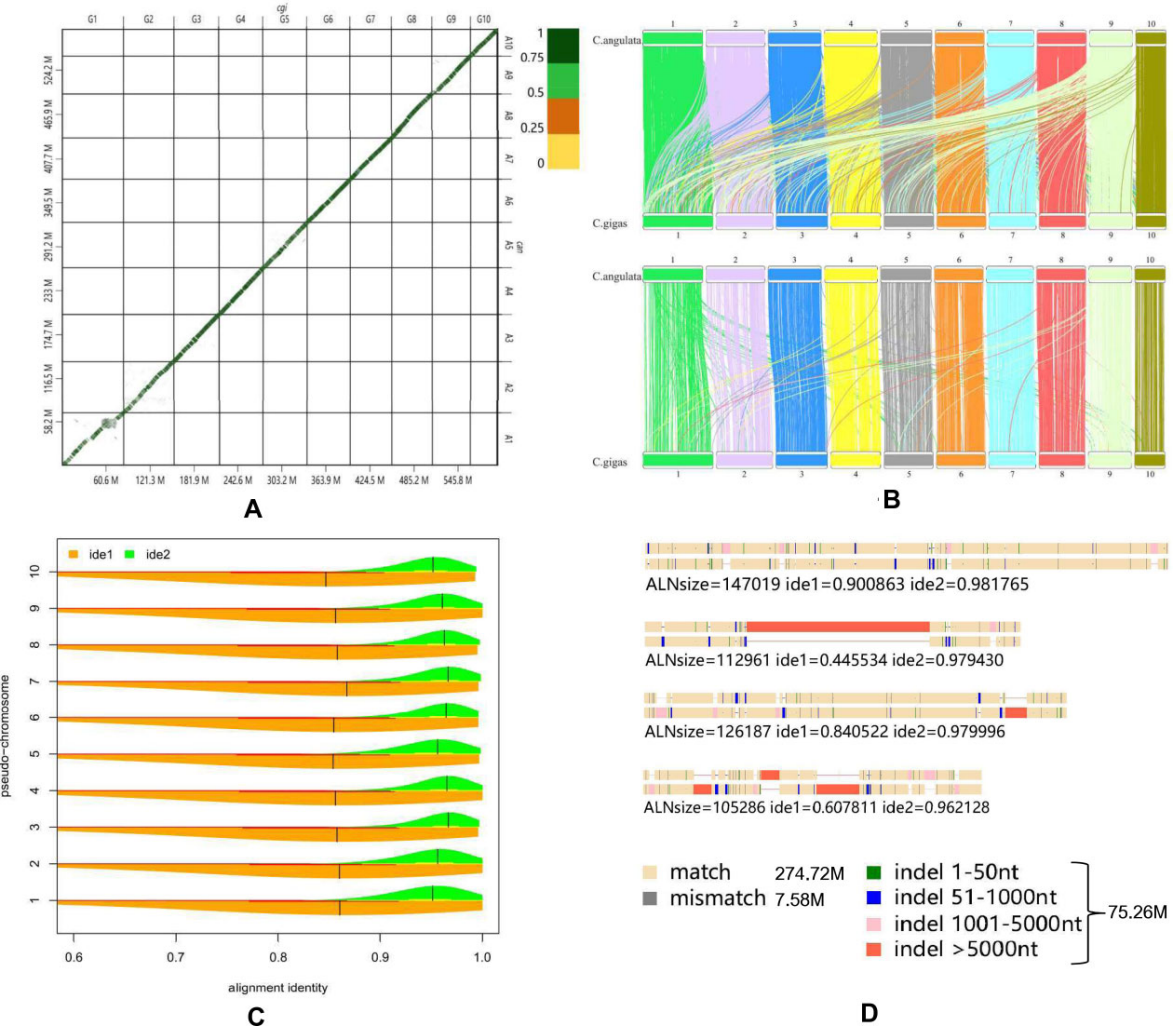
The direct DNA sequence comparison between *C. angulata* and *C. gigas* genomes. (A) The dot plot for the comparison of the 10 pairs of pseudo-chromosome sequences. Top x-axis: G1–G10, the pseudo-chromosomes (10 largest scaffolds: cgiscf1–cgiscf10) in the *C. gigas* genome. Right y-axis: A1–A10, the pseudo-chromosomes (10 largest scaffolds: canscf1–canscf10) in the *C. angulata* genome. Bottom x-axis: the length of G1–G10. Left y-axis: the length of A1–A10. The sequence identities were represented by the upper right colors. (B) The synteny of conserved DNA blocks between the two genomes. The two images were drawn from DNA blocks with a size of ≥1,000 bp and ≥10,000 bp (max = 226,631 bp), respectively. (C) The distribution of alignment identities of the conserved DNA blocks in the 10 pairs of pseudo-chromosomes. x-axis: sequence alignment identity. y-axis: the 10 pseudo-chromosome pairs. The black vertical lines in the bean plot denote the median values. ide1: the alignment identity measured by the gap-uncompressed method. ide2: the alignment identity measured by the gap-compressed method. (D) A sketch map for the large indels in the alignments of conserved DNA segments. The four DNA alignments are from A3:13,369,498–13,506,783 vs G3:12,755,799–12,900,025, A5:4,508,362–4,563,006 vs G5:5,295,108–5,404,622, A6:20,475,358-20,598,634 vs G6:21,302,015–21,412,762, and A7:6,838,813–6,932,940 vs G7:7,304,889–7,382,157, respectively.

A direct comparison of the DNA sequences of the two organisms revealed an overall pairwise alignment identity of greater than 0.75 at the whole-genome level (Fig. 3A). Although repeats (such as the widespread interspersed repetitive elements) could lead to alignments at multiple positions, similar DNA fragments in the 10 pseudo-chromosomes of *C. angulata* with its counterparts of *C. gigas* constituted most of the larger conserved DNA sequence block pairs. This implies significant synteny and high genomic similarity between the two assemblies (Fig. 3A, B). Detailed parsing of the alignments of conserved segments in the 10 pairs of pseudo-chromosomes found that the BLAST identity medians (means) were 0.85 to 0.87 (0.72–0.80), whereas the gap-compressed identity medians (means) were 0.95 to 0.97 (0.96–0.97) (Fig. 3C). The total alignment, match, mismatch, and indel sizes were about 357.6 M, 274.7 M, 7.58 M, and 75.26 M, respectively (Fig. 3D). Moreover, the average sequence divergence rates calculated by gap-uncompressed and gap-compressed methods were 0.232 and 0.031, respectively. It is obvious that gaps (indels) can cause a much larger number of alignment differences in the calculation of sequence identity (Fig. 3D). The larger indels of ≥50 bp (usually considered one kind of structural variation) had a total size of 51.36 M, accounting for 68.3% of the total indel length. Thus, the divergence rates of the two genomes estimated by nucleotide substitution (mismatch), small indels (gaps, <50 bp) and big indels (gaps, ≥50 bp) were 0.021, 0.067, and 0.144, respectively. This indicates that structural variations such as deletions and insertions were the major sources leading to the genomic divergences of *C. angulata* and *C. gigas*.

**Figure 4:**
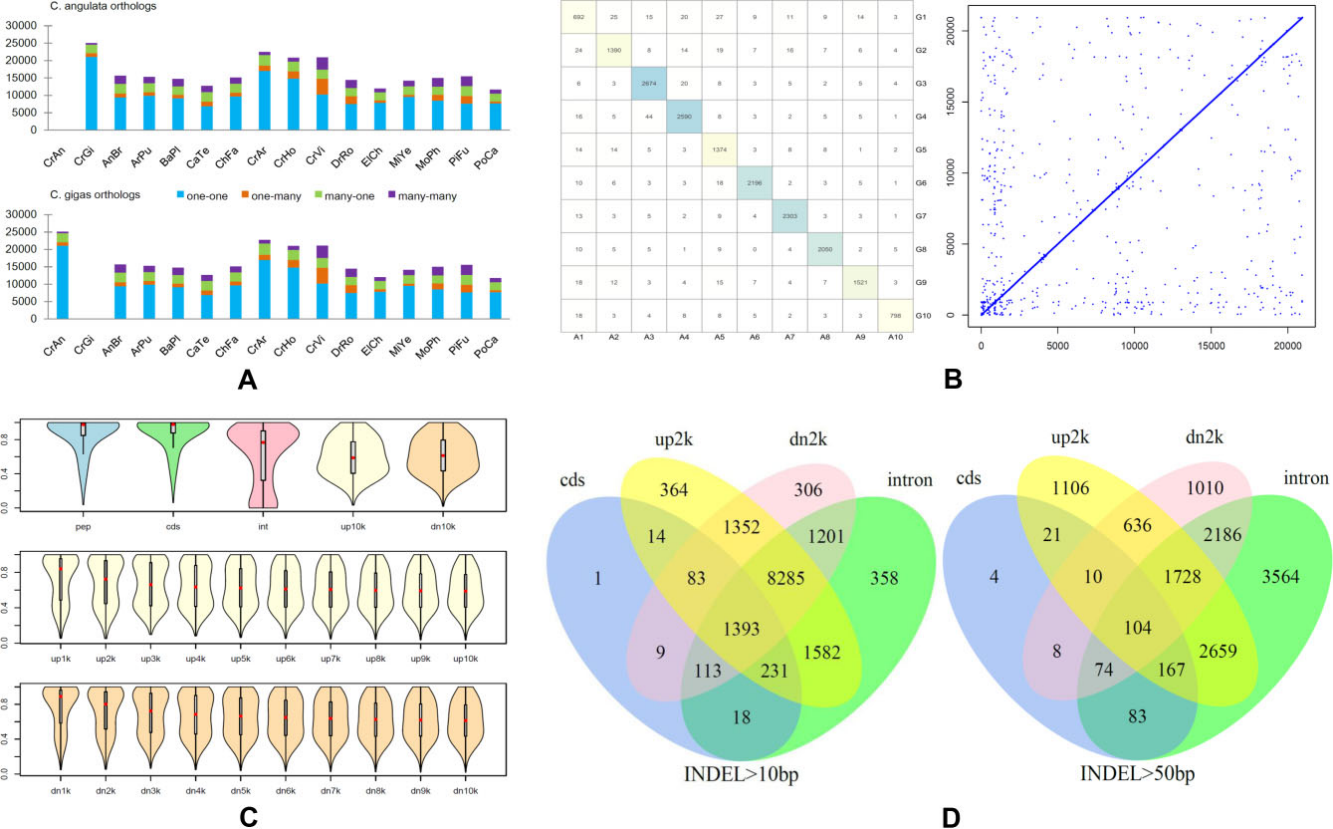
Orthologous genes comparison between *C. angulata* and *C. gigas* genomes. (A) The number of orthologs of 4 subtypes in 15 genomes. x-axis: the species names. y-axis: the number of genes. (B) The orthologous genes between the two genomes. Left: the number distribution of orthologous genes in the 10 pseudo-chromosome pairs. A1–A10, the pseudo-chromosomes (10 largest scaffolds: canscf1–canscf10) in the *C. angulata* genome. G1–G10, the pseudo-chromosomes (10 largest scaffolds: cgiscf1–cgiscf10) in the *C. gigas* genome. Right: the dot plot for genomic position orders of the orthologs. x-axis: *C. angulata* gene orders. y-axis: *C. gigas* gene orders. (C) The violin plot for sequence identities of different gene regions of orthologous genes. Top: the identities of protein sequences (“pep”), coding sequences (“cds”), introns (“int”), upstream 10-kbp (“up10k”) segments, and downstream 10-kbp (“dn10k”) segments. Middle: the identities of “up1k” to “up10k” segments. Bottom: the identities of “dn1k” to “dn10k” segments. (D) The number of orthologous gene pairs that contained indels in different gene regions. Left: indel size >10 bp. Right: indel size >100 bp.

The construction of orthologous groups of two or more genomes underlies comparative and phylogenetic analyses of gene sets at the coding gene level. A total of 34,043 orthologous groups were identified in *C. angulata* and *C. gigas* together with 14 other genomes. From the comparison between the two genomes, the number of orthologous genes in the 4 subtypes (i.e., one-to-one, one-to-many, many-to-one, and many-to-many orthologs) were 21,055, 1,080, 2,427, and 484 and 21,055, 1,013, 2,579, and 477 in the *C. angulata* and *C. gigas* genomes, respectively (Fig. 4A). Most of these orthologs were located in the corresponding pseudo-chromosome pairs in the two genomes, and the genomic position orders of the one-to-one orthologs were strongly correlated (Spearman's rank correlation rho = 0.966, *P* < 1.0e-10). This suggests a distribution pattern characterized by highly conserved spatial collinearity (Fig. 4B). The level of sequence conservation varied dramatically across different gene regions. The average coding sequences (“cds”), deduced protein sequences (“pep”), introns (“int”), upstream 10-kbp segments (“up10k”), and downstream 10-kbp segments (“dn10k”) identities of the orthologous gene pairs of the two genomes were 0.8894, 0.8776, 0.6156, 0.6032, and 0.6215, respectively. The average sequence identities of the upstream and downstream regions gradually decreased with increasing distance from the CDS (Mann–Kendall trend test, *P* < 1.0e-4) (Fig. 4C). Gene structure (referring to the number of CDS) had an impact on sequence identities. The average “cds” (“pep,” “int,” “up10k,” “dn10k”) identities of 21,012 gene pairs with identical gene structure were 0.9479 (0.9405, 0.6881, 0.6371, 0.6516) and were significantly higher than those of 5,847 gene pairs (0.6791 (0.6517, 0.3554, 0.4811, 0.5135)) with different gene structure (Welch two-sample *t*-test, *P* < 1.0e-10). There were a total of 15,475 highly conserved ortholog pairs, which shared identical gene structure, and both the “cds” and the “pep” identities were bigger than 0.90. A further investigation on the alignments of the 15,475 gene pairs revealed that the indels located in the intron, “up-2k,” and “dn2k” were the major elements leading to the sequence divergence of orthologous gene regions. The number of ortholog pairs that harbored indels with a size of >10 bp in the CDS, intron, “up-2k,” and “dn2k” regions were 1,862, 13,181, 13,304, and 12,742, respectively (Fig. 4D). The number of ortholog pairs that harbored indels with a size of >50 bp in the CDS, intron, “up-2k,” and “dn2k” regions were 471, 10,565, 6,431, and 5,756, respectively.

**Figure 5:**
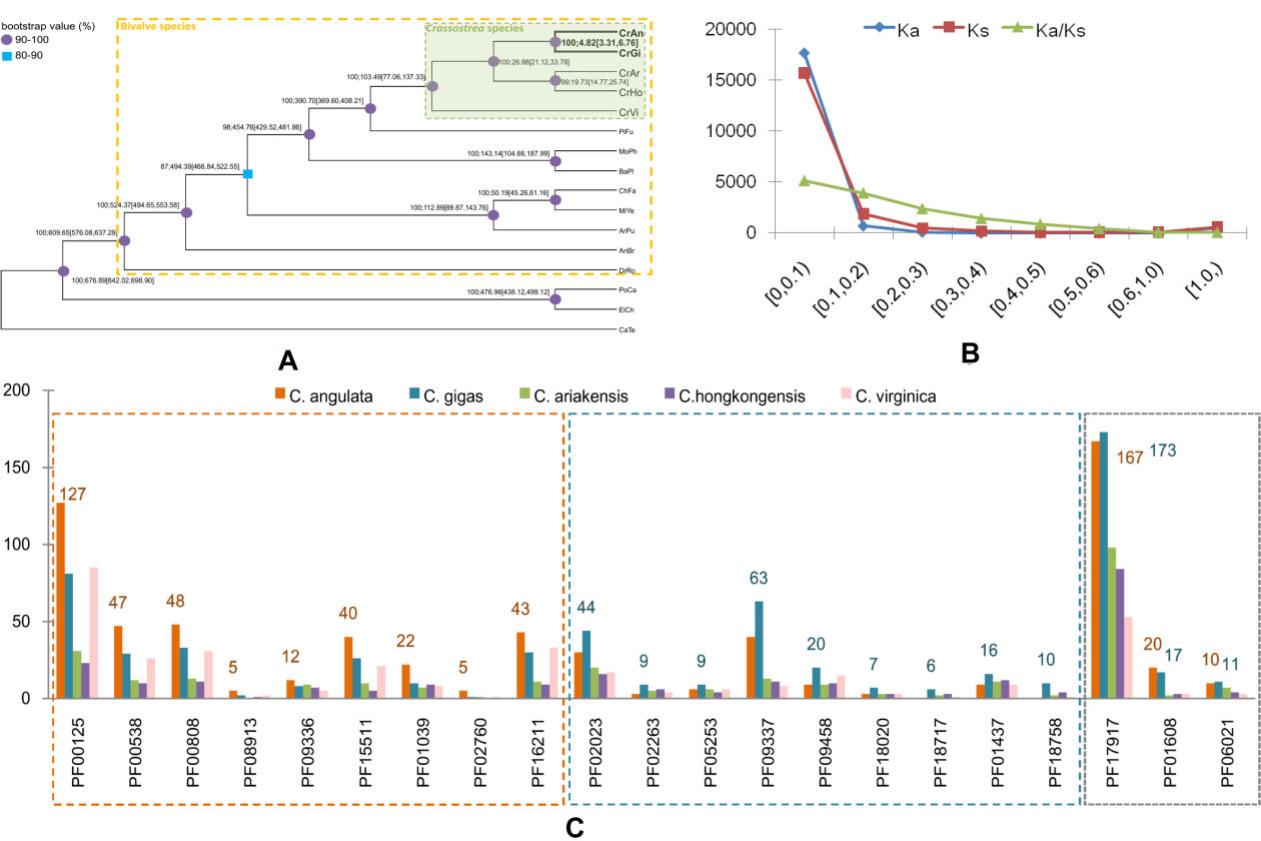
Phylogenetic and evolutionary analysis. (A) The phylogenetic tree and divergence time of several *Crassostrea* species. The node label numbers denote the bootstrap value (percent), the estimated mean divergence time (MYA), and 95% confidence interval (in square brackets). (B) The distribution of Ka, Ks, and Ka/Ks values. (C) The protein domain abundance analysis. Left box: expanded in *C. angulata*; Middle: expanded in *C. gigas*; Right: expanded in *C. angulata* and *C. gigas*.

Based on the orthologous gene inference of multiple species, 519 single-copy genes were identified and submitted for the construction of a species phylogenetic tree. *C. angulata* and *C. gigas* were first clustered into a clade, and their divergence time was estimated to be 4.82 MYA (95% confidence interval, 3.31–6.76)(Fig. 5A). This was far shorter than the divergence times among other *Crassostrea* species. Ka/Ks analysis of the orthologs of *C. angulata* and *C. gigas* revealed that the vast majority of Ka and Ks values were less than 0.1, most of the Ka/Ks values were below 0.4, and only 17 gene pairs had Ka/Ks values greater than 1 (Fig. 5B). Gene annotations showed that only 6 of the 17 genes had SWISS-PROT matches and that the NR matches of the remaining 11 genes were mostly uncharacterized proteins (Supplementary Table S4). The average transcripts per million (TPM) values of 11 of the 18 genes were greater than 2.0 in the gills under normal physiological conditions. Protein domain abundance analysis revealed 21 expanded protein domains, of which 9 were in *C. angulata*, another 9 were in *C. gigas*, and 3 were in both species (Fig. 5C; Supplementary Table S5). In *C. angulata*, the “Histone”-related domains were significantly expanded: there were 127 genes with the “Histone” domain (PF00125: core histone H2A/H2B/H3/H4) and 47 genes with the “Linker_histone” domain (PF00538: linker histone H1 and H5 family). The maximum GNs of the 2 protein domains in other species were 85 and 29, respectively. *C. angulata* had 22 genes with the “Carboxyl_trans” domain (PF01039: Carboxyl transferase domain), whereas *C. gigas* had 10 and other species only had 3 to 9. In *C. gigas*, the GN of “zf-H2C2” (PF09337: H2C2 zinc finger) domain was 63, which was much higher than the GN of 40 in *C. angulata* and 0 to 23 in other species. The “H_lectin” (PF09458: H-type lectin domain), “SCAN” (PF02023: SCAN domain), and “KDZ” (PF18758: Kyakuja–Dileera–Zisupton transposase) domains were also enriched in *C. gigas*. Only 3 domains (PF17917: RNase H-like domain found in reverse transcriptase; PF01608.18: I/LWEQ domain; and PF06021: Aralkyl acyl-CoA:amino acid N-acyltransferase) were overrepresented in both organisms.

**Figure 6:**
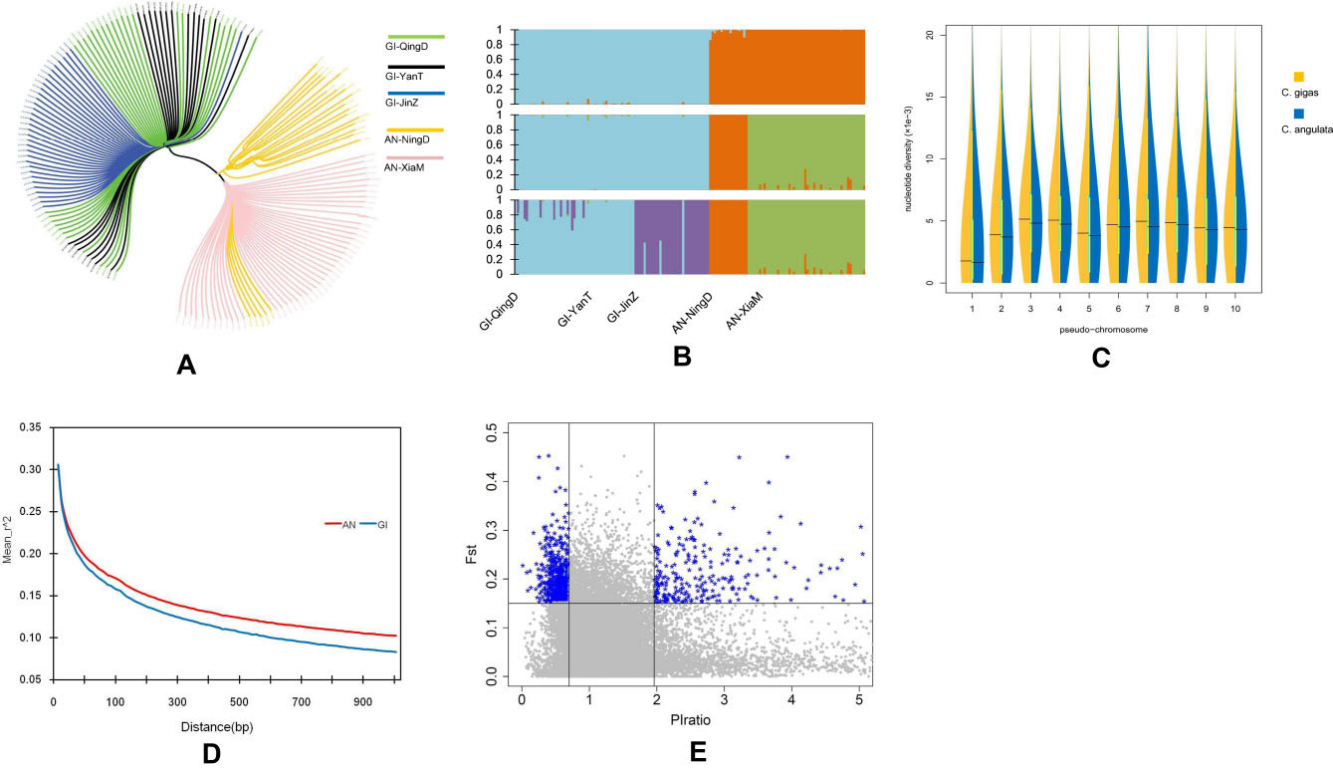
Population analysis based on resequencing data of *C. angulata* and *C. gigas*. (A) The phylogenetic tree of oysters from 5 populations. GI-QingD: *C. gigas* oysters of Qingdao. GI-YanT: *C. gigas* of Yantai. GI-JinZ: *C. gigas* of Jinzhou. AN-NingD: *C. angulata* of Ningde. AN-XiaM: *C. angulata* of Xiamen. (B) The structure inference analysis. The 3 images refer to the admixture proportions at K = 2, K = 3, and K = 4, respectively. (C) The distribution of nucleotide diversity of the 10 pseudo-chromosome sequences. (D) Linkage disequilibrium decay curves of the two populations. AN: *C. angulata*; GI: *C. gigas*. (E) The distribution of *F*st and θπ ratio divergence. The blue dots on the upper left denote the genomic regions with largest *F*st (>0.152, top 5%) and θ_π_ ratio (<0.70, bottom 5%); the blue dots on the upper right denote the genomic regions with largest *F*st (>0.152, top 5%) and θ_π_ ratio (>1.96, top 5%).

Based on the resequencing data from 69 *C. angulata* and 86 *C. gigas* oysters, a total of 15.1 M high-confidence biallelic SNPs with minor allele frequency (MAF) >0.01 and missing rate <0.05 were identified. The two species were clearly clustered into two large groups based on phylogenetic analysis and population structure inference (Fig.   6A, B). Within each species, genetic admixing was observed in some individuals from different locations, and the oysters could not be fully separated based on their sampling locations. At the whole-genome level, the nucleotide diversity (θ_π_) of *C. gigas* and *C. angulata* was 4.13 × 10^−3^ and 3.94 × 10^−3^, respectively, and the former was slightly higher than the latter. At each of the 10 pseudo-chromosomes, the θ_π_ of *C. gigas* was also slightly higher than that of *C. angulata* (Kolmogorov–Smirnov test, *P* < 0.05) (Fig. 6C). Rapid linkage disequilibrium decay was observed for both species, and the *r*^2^ values decreased from 0.3 to 0.15 within a 200- to 300-bp span (Fig. 6D).

For 93.1% of the SNPs, the allele frequency difference between the two populations was less than 0.30. A total of 82,245 species-enriched and 2,756 species-specific SNPs had allele frequency differences greater than 0.75 and 0.95. The proportion of species-enriched “intergenic-,” “downstream-,” “upstream-,” “intron-,” “synonymous-,” and “nonsynonymous-” SNPs in the total SNPs of the above 6 types were 0.007474, 0.007022, 0.006609, 0.007196, 0.006716, and 0.008383, respectively. The proportions of species-specific SNPs among the above 6 types were 0.000265, 0.000207, 0.000244, 0.000218, 0.000188, and 0.000325, respectively. In both cases, the proportion of nonsynonymous SNPs was significantly higher than that of other types of SNPs (chi-square tests, *P* < 0.05).

The *F*st median of the two populations estimated by the 10-kbp sliding windows was 0.055. Additionally, the genomic regions with the largest *F*st (>0.152, top 5%) and θ_π_ ratio divergence (<0.70 or >1.96; bottom and top 5%) was around 13.2 Mbp, overlapping 1,088 coding genes (Fig. 6E). In total, 704 and 384 putative genes under selection were identified in *C. angulata* and *C. gigas*, respectively (Supplementary Table S6). In *C. angulata*, these genes were enriched in 25 pathways, including the cGMP-PKG signaling pathway, pentose phosphate pathway, fat digestion and absorption, protein digestion and absorption, and the HIF-1 signaling pathway. In *C. gigas*, genes were enriched in 7 pathways, including protein digestion and absorption, ovarian steroidogenesis, and progesterone-mediated oocyte maturation (Supplementary Table S7). In addition, selection signals were detected in two heat shock 70-kDa protein (HSP70) genes and one HSP90 gene in *C. angulata* and *C. gigas*.

## Discussion

Because of their considerable roles in aquatic ecological systems and as food or industrial materials for humans, mollusks have attracted more research attention than ever, and high-quality genomes have gradually become a necessary resource for basic research. It has become common for distinct research groups to publish genome assemblies for the same species or release several genomes simultaneously for different species [9, 10, 15, 16, 18, 19, 62]. Multiple genomes of the same organism once seemed unnecessary when sequencing was too expensive and a standard reference was sufficient for most analyses. However, they are now considered essential in an era when costs are dramatically reduced and more focus is paid to the exploration of different levels of genomic variations in the scenario of a pan-genome framework [63]. Here, we provided genomes for two closely related *Crassostrea* oyster congeners, the Portuguese oyster *C. angulata* and the Pacific oyster *C. gigas*. We performed comparative studies at the single-genome and population levels, which presented improved assembly qualities and may further deepen our understanding of oyster genome diversities.

Oysters and other bivalves have high levels of genomic polymorphisms [5, 64], which are the main barriers to a high-quality assembly. A traditional method to reduce heterozygosity is inbreeding, but it is quite difficult to obtain individuals with a high inbreeding coefficient and maintain multiple-generation inbreeding strains in bivalves; this strategy has been applied to genome projects only in a few species, such as the Pacific oyster [6] and Yesso scallop [65]. Even assisted by the fosmid-pooling hierarchical assembly approach, the first version of the Pacific oyster genome was fragmented, with a contig N50 of several kilobases and a scaffold N50 of several hundred kilobases, which was of the same order of magnitude as the later-appearing genomes of other bivalves produced by similar sequencing strategies. High heterozygosity and repetitive sequences can result in redundancy and imperceptible assembly errors in contigs [66, 67]. Long DNA reads spanning repeats are key to maximizing genome quality. Based on long DNA reads and Hi-C scaffolding, nearly all bivalves genomes released in the past 2 to 3 years were at the chromosome level with contig N50 >1 million bases. BUSCO evaluation indicated that several of the previously published *Crassostrea* oyster genomes had higher completeness (C >95%), but none of them had a result of “S >95%, D <1%, F <1%, M <1%,” and most of the duplicated BUSCOs were >2.5% (Supplementary File 3), implying possible redundancies. In the present study, BUSCOs of the two genomes reached “S >98%, D <1%, F <1%, M <1%” and contig N50 were both >5.0 M (*C. angulata* >12 M), demonstrating an significant improvement in basic assembly quality assessment metrics.

The *C. angulata* and *C. gigas* genomes were chromosome level and fully haplotype resolved, which is the most typical feature of these two genomes. To date, complete phased genomes have only been accomplished in several species [20, 23, 24], although the trend for building phased genomes and their advantages in related studies have been widely accepted. A major challenge is the lack of global phase information for separating haplotypes over long genomic distances [24]. The trio-binning strategy can group the sequencing reads of a diploid genome by leveraging parent-specific *k*-mers, thus simplifying the haplotype assembly [26]. A basic requirement for read binning is the isolation of an adequate number of parent-specific *k*-mers [68]. In this study, a high proportion of unique *k*-mers of 4 lengths could effectively separate more than 97% of the CH1 long reads and 61% to 91% of the short DNA reads of Hi-C and WGS. This suggests that trio binning could turn the high-heterozygosity disadvantage that once hindered genome assembly into a distinct advantage. The present study demonstrated that the trio-binning strategy is an effective approach for building haplotype-derived bivalve genomes.

The two genomes had similar features in terms of GC content, repeat content, coding gene numbers and sizes, and gene annotations, which were comparable to those of other *Crassostrea* genomes. However, their heterozygosity was significantly higher than that of the Jinjiang and Hong Kong oysters, as estimated by *k*-mer or resequencing analysis [16, 17]. A direct comparison of the two genomes revealed a large number of conserved DNA sequence block pairs and an average gap-compressed identity of greater than 0.96, implying high similarity and significant synteny of the two genomes. Many studies have used the *C. gigas* genome as a reference to align *C. angulata* sequencing reads for subsequent analysis [56, 69]. Considering the read mapping rate, the difference was negligible. In contrast, large insertions and deletions were common between the two genomes and could lead to a divergence rate of 0.144, indicating that structural variations were the major elements that varied between the two genomes. Structural variations are an important source of genetic diversity [70], and many copy number variations have been reported in the Pacific oyster *C. gigas* and the eastern oyster *C. virginica* genomes [10, 71, 72].

In the genus *Crassostrea*, the divergence time of *C. angulata* and *C. gigas* was 4.82 MYA, even though they were the most closely related species. This is greater than the 2.72 MYA estimated by mitochondrial genes [50]. This may be because 209 single-copy genes were used in the present study, whereas only 12 coding genes were used in the previous study. A total of 21,055 one-to-one ortholog gene pairs were identified, as well as 15,475 shared identical gene structures, >90% identity in coding and protein sequences, and highly conserved spatial collinearity. This could largely explain the previous report that found high macro-collinearity and the same order of most of the transferable expressed sequence tag (EST) markers in *C. angulata* and *C. gigas* genomes [73]. The low Ks value and Ka/Ks ratios suggested that most of the orthologous genes were conserved between the two genomes and were subject to strong selective constraints [53].

*C. gigas* and *C. angulat*a have similar external morphological features, hybridize under natural conditions, and produce fertile offspring [3, 74, 75]. Previous studies have suggested that *C. angulata* was a subspecies of *C. gigas* [1]. The significant synteny and high genomic similarity of the two assemblies, the large number of highly conserved ortholog gene pairs, and population analysis in this study could provide novel evidences supporting this view. Although both species had large intra- and interindividual polymorphisms, *F*st estimation indicated that most of the genomic regions showed low to moderate levels of genetic differentiation, which is in accordance with our previous report [69]. An increasing number of studies have found physiological differences between the two species in terms of growth, thermal tolerance, fatty acid content and composition [3, 12, 76], and adaptive divergence of plasticity in environmentally responsive genes [77]. In the present study, 1,088 coding genes were identified as candidate genes possibly under selection. These genes included HSP70 and HSP90, which are key molecules in protein homeostasis, thermal adaptation, and stress response [78]. Genes related to fat and protein digestion and absorption were enriched, which was in accordance with our previous work showing that energy metabolism plays a considerable role in the formation of adaptive traits in the two species [12, 13]. These genes could provide new resources for understanding the evolution and connections between genes and biological features of the two congeneric oyster species.

## Conclusion

Two chromosome-level fully phased genomes were constructed for the Portuguese oyster *C. angulata* and the Pacific oyster *C. gigas* through a trio-binning strategy. They were characterized by high BUSCO completeness and contig N50 size and ranked at the top of marine invertebrate genomes with high contiguity and integrity. The general features of the two genomes were similar and 15,475 highly conserved orthologous gene pairs were identified. At the population level, individuals of the two species were clearly clustered into two large groups; 2,756 species-specific SNPs and 1,088 coding genes, possibly under selection, were identified. The study provides novel data resources that contribute to the genomics, genetics, and evolution studies in mollusks.

## Supplementary Material

giad077_GIGA-D-23-00117_Original_Submission

giad077_GIGA-D-23-00117_Revision_1

giad077_Response_to_Reviewer_Comments_Original_Submission

giad077_Reviewer_1_Report_Original_Submission Vanessa Gonzalez, Ph.D. -- 6/23/2023 Reviewed

giad077_Reviewer_2_Report_Original_Submission Marcela Uliano-Silva -- 7/12/2023 Reviewed

giad077_Supplemental_Files

## Data Availability

The genomes and raw sequencing reads produced in the study have been released in the NCBI database. Genome assemblies: *C. angulata*, GCA_025765675.3; *C. gigas*, GCA_025765685.3. Sequencing reads: paternal *C. angulata*, SRR21185640; maternal *C. gigas*, SRR21185639; the hybrid offspring, SRR21185636, SRR21185637, SRR21185638; 47 newly resequenced *C. angulata* oysters: SRR22668975–SRR22669021. All supporting data are available in the *GigaScience* GigaDB database [79].

## Additional Files

**Supplementary File 1**. Notes for the usage of resequencing data.

**Supplementary File 2**. The control file for the running of MCMCTree program in the PAML package.

**Supplementary File 3**. The BUSCOs of the several *Crassostrea* oyster genomes.

**Supplementary Table S1**. The genomes used for ortholog group construction.

**Supplementary Table S2**. Genome assessment by short DNA reads and transcriptome mapping.

**Supplementary Table S3**. The repeat contents of the two genomes.

**Supplementary Table S4**. The gene pairs with Ka/Ks >1.

**Supplementary Table S5**. The expanded gene families defined by PFAM domains.

**Supplementary Table S6**. The putative genes under selection in *C. angulata* and *C. gigas*.

**Supplementary Table S7**. The enriched KEGG pathways of the genes under selection.

## Abbreviations

BLAST: Basic Local Alignment Search Tool; bp: base pairs; BQ: base quality; BUSCO: Benchmarking Universal Single-Copy Orthologs; C: complete; D: completely duplicated; F: fragmented; GN: gene number; GO: Gene Ontology; HiFi: high-fidelity; kbp: kilobase pairs; KEGG: Kyoto Encyclopedia of Genes and Genomes; M: missing; Mbp: megabase pair; NCBI: The National Center for Biotechnology Information; NR: nonredundant; PN: pass number; RNA-seq: RNA sequencing; RQ: read quality; S: complete and single-copy; TE: transposable element; TRF: Tandem Repeats Finder.
