# Supplementary material for: Construction and analysis of the chromosome-level haplotype-resolved genomes of two Crassostrea oyster congeners: Crassostrea angulata and Crassostrea gigas
Source: Gigascience. 2023 Oct 3;12:giad077. doi: 10.1093/gigascience/giad077 (PMC10546077; doi:10.1093/gigascience/giad077)
Supplement: giad077_GIGA-D-23-00117_Original_Submission [file giad077_giga-d-23-00117_original_submission.pdf]

GigaScience

Construction and analysis of the chromosome-level haplotype-resolved genomes of two Crassostrea oyster congeners: Crassostrea angulata and C. gigas

--Manuscript Draft--

**Manuscript Number:**	GIGA-D-23-00117
**Full Title:**	Construction and analysis of the chromosome-level haplotype-resolved genomes of two Crassostrea oyster congeners: Crassostrea angulata and C. gigas
**Article Type:**	Data Note
**Funding Information:**	01 the National Key R&D Program of China (2022YFD2400301) — Not applicable<br>02 the National Natural Science Foundation of China (41876169) — Not applicable<br>03 the Key Research and Development Program of Shandong (2022LZGC015) — Not applicable<br>04 the Earmarked Fund for China Agriculture Research System (No. CARS-49) — Not applicable
**Abstract:**	Background: The Portuguese oyster Crassostrea angulata and the Pacific oyster C. gigas are two major Crassostrea species that are naturally distributed along the Northwest Pacific coast and possess great ecological and economic value. Here, we report the construction and comparative analysis of the chromosome-level haplotype-resolved genomes of two oyster congeners.<br>Findings: Based on a trio-binning strategy, the PacBio high-fidelity and Illumina Hi-C reads of the offspring of the hybrid cross C. angulata (♂) × C. gigas (♀) were partitioned and independently assembled to construct two chromosome-level fully phased genomes. The assembly size (contigN50 size, BUSCO completeness) of the two genomes were 582.4 M (12.8 M, 99.1%) and 606.4 M (5.46 M, 98.9%) for C. angulata and C. gigas, respectively, ranking at the top of mollusc genomes with high contiguity and integrity. The general features of the two genomes were highly similar, and 15,475 highly conserved ortholog gene pairs shared identical gene structures and similar genomic locations. Highly similar sequences can be primarily identified in the coding regions, whereas most non-coding regions and introns of genes in the same ortholog group contain substantial small genomic and/or structural variations. Based on population resequencing analysis, a total of 2,756 species-specific SNPs and 1,088 genes possibly under selection were identified.<br>Conclusions: This is the first report on high-quality chromosome-level fully phased genomes in marine invertebrates. The study provides fundamental resources for the research on mollusc genetics, comparative genomics and molecular evolution.
**Corresponding Author:**	Haigang Qi<br>Institute of Oceanology Chinese Academy of Sciences<br>Qingdao, CHINA
**Corresponding Author Secondary Information:**	
**Corresponding Author's Institution:**	Institute of Oceanology Chinese Academy of Sciences
**Corresponding Author's Secondary Institution:**	
**First Author:**	Haigang Qi
**First Author Secondary Information:**	
**Order of Authors:**	Haigang Qi<br>Rihao Cong<br>Yanjun Wang

Powered by Editorial Manager® and ProduXion Manager® from Aries Systems Corporation

	Li Li
	Guofan Zhang
**Order of Authors Secondary Information:**	
**Additional Information:**	
**Question**	**Response**
Are you submitting this manuscript to a special series or article collection?	No
**Experimental design and statistics**<br><br>Full details of the experimental design and statistical methods used should be given in the Methods section, as detailed in our Minimum Standards Reporting Checklist. Information essential to interpreting the data presented should be made available in the figure legends.<br><br>Have you included all the information requested in your manuscript?	Yes
**Resources**<br><br>A description of all resources used, including antibodies, cell lines, animals and software tools, with enough information to allow them to be uniquely identified, should be included in the Methods section. Authors are strongly encouraged to cite Research Resource Identifiers (RRIDs) for antibodies, model organisms and tools, where possible.<br><br>Have you included the information requested as detailed in our Minimum Standards Reporting Checklist?	Yes
**Availability of data and materials**<br><br>All datasets and code on which the conclusions of the paper rely must be either included in your submission or deposited in publicly available repositories (where available and ethically appropriate), referencing such data using	Yes

a unique identifier in the references and in the “Availability of Data and Materials” section of your manuscript.<br><br>Have you have met the above requirement as detailed in our Minimum Standards Reporting Checklist?	

Powered by Editorial Manager® and ProduXion Manager® from Aries Systems Corporation

Manuscript
Click here to access/download;Manuscript;GIGA-D-23-00117-0518f1.docx

Construction and analysis of the chromosome-level haplotype-resolved genomes of two *Crassostrea* oyster congeners: *Crassostrea angulata* and *C. gigas*

Haigang Qi[1,2,5], Rihao Cong[1,2,5], Yanjun Wang[3], Li Li[1,4,5*], Guofan Zhang[1,2,4*]

1 CAS and Shandong Province Key Laboratory of Experimental Marine Biology, Center for Ocean Mega-Science, Institute of Oceanology, Chinese Academy of Sciences, Qingdao, China
2 Laboratory for Marine Biology and Biotechnology, Qingdao National Laboratory for Marine Science and Technology, Qingdao, China
3 Marine Science Data Center, Institute of Oceanology, Chinese Academy of Sciences, Qingdao, China
4 National and Local Joint Engineering Laboratory of Ecological Mariculture, Qingdao, China
5 Shandong Technology Innovation Center of Oyster Seed Industry, Qingdao, China
*Corresponding author: lili@qdio.ac.cn; gfzhang@qdio.ac.cn

Abstract

Background: The Portuguese oyster *Crassostrea angulata* and the Pacific oyster *C. gigas* are two major *Crassostrea* species that are naturally distributed along the Northwest Pacific coast and possess great ecological and economic value. Here, we report the construction and comparative analysis of the chromosome-level haplotype-resolved genomes of two oyster congeners.

Findings: Based on a trio-binning strategy, the PacBio high-fidelity and Illumina Hi-C reads of the offspring of the hybrid cross *C. angulata* (♂) × *C.* gigas (♀) were partitioned and independently assembled to construct two chromosome-level fully phased genomes. The assembly size (contigN50 size, BUSCO completeness) of the two genomes were 582.4 M (12.8 M, 99.1%) and 606.4 M (5.46 M, 98.9%) for *C. angulata* and *C. gigas*, respectively, ranking at the top of mollusc genomes with high contiguity and integrity. The general features of the two genomes were highly similar, and 15,475 highly conserved ortholog gene pairs shared identical gene structures and similar genomic locations. Highly similar sequences can be primarily identified in the coding regions, whereas most non-coding regions and introns of genes in the same ortholog group contain substantial small genomic and/or structural variations. Based on population resequencing analysis, a total of 2,756 species-specific SNPs and 1,088 genes possibly under selection were identified.

Conclusions: This is the first report on high-quality chromosome-level fully phased genomes in marine invertebrates. The study provides fundamental resources for the research on mollusc genetics, comparative genomics and molecular evolution.

Keywords: oyster, *Crassostrea angulata*, *Crassostrea gigas*, trio-binning, haplotype-resolved genome

Introduction

Mollusca is the second largest phylum in the animal kingdom and contains the highest number of marine invertebrates. Oysters are filter-feeding bivalves belonging to the family Ostreidae. They are widely distributed in shallow seas and estuaries and constitute an essential component of marine ecosystems. Oysters in the genus *Crassostrea* are of special significance, as they can grow together by settling on each other's shells and forming massive reefs, which are similar to coral reefs in terms of their ecological importance. With a long history as a human food source, oysters play a considerable role in the fishery and aquaculture industries. The Pacific oyster *C. gigas* (Thunberg, 1793) and Portuguese oyster *C. angulata* (Lamarck, 1819) are two dominant *Crassostrea* species. They are known as cupped oysters, naturally inhabiting the Northwest Pacific coasts. In China, *C. gigas* is found on the northern coast of the Yangtze Estuary, whereas *C. angulata* is found in the south of the Yangtze Estuary [1]. Although *C. angulata* was first identified and named in Portugal, early studies proved that European *C. angulata* originated from Asia [2-4] and could be considered a subspecies of *C. gigas* . In China and some recent studies, *C. angulata* was often called the Fujian oyster, and a recommendation for renaming it to *C. gigas angulata* has been proposed [1]. As our focus was not on the oyster taxonomy, in the study we still use the words 'two species' to refer to them. Their annual production has reached 4.0 million tons since 2004 [5] and they have been the oyster species with the highest consumption and trade volume.

C. gigas was considered a model organism in Lophotrochozoa [5] and among the first batch of mollusk species with an accessible whole genome assembly [6]. As a first release, the *C. gigas* genome version ‘v9’ (GenBank Acc No: GCA_000297895.1) has brought a substantial effect on oyster basic research. However, due to technical limitations, the ‘v9’ assembly was highly fragmented and contained some assembling or annotation errors [7,8], despite the use of an oyster derived from four generations of full-sibling mating and a fosmid-pooling hierarchical assembly strategy. Two chromosome-level *C. gigas* genomes have recently been published [9,10], which have improved the assembly quality and further expanded the genomic resources for the research community. Several studies have been conducted to explore the differentiation of the two species [11-13], but the *C. angulata* genome is yet to be available, and a full comparison with *C. gigas* at the whole-genome sequence level is lacking. This, to some degree, limits our understanding of the genomics and evolution of *Crassostrea* oysters..

Before 2015, only a few mollusk genomes were published. With the rapid development of sequencing and scaffolding technologies, it is feasible to complete chromosome-level genome assembly at a relatively low cost for non-model organisms. The number of mollusk genomes started to explode in 2017 [14]; in the past 1-3 years, chromosome-level genomes of Ostreidae oysters have been massively released, including the Pacific oyster *C. gigas* [9,10], the Jinjiang oyster *C. ariakensis* [15,16], the Hong Kong oyster *C. hongkongensis* [17] and the European flat oyster *Ostrea edulis* [18,19]. These genomes have covered most of the oyster species that are of great ecological and economic value throughout the world.

A trend for genome assembling is to construct the haplotype-resolved (phased) sequences, which are more favorable for variations discovery and genetic dissection of complex traits than the traditional "squashed" or "mosaic" genomes [20,21]. Phasing in highly divergent regions can be achieved by utilizing SNP allele linkage information through a variety of programs [22]. However, building a fully phased genome is far more challenging. The complete high-quality haplotype-resolved

genomes have been accomplished in several species in the last few years [20,21,23,24]. This is largely ascribed to the advent of technology producing highly accurate long DNA sequences represented by the PacBio high-fidelity (HiFi) reads, in that the once-widely used long reads are noisy with 10–15% error rates. Additionally, phasing was usually lost after corrections [25]. The development of assembly methods or algorithms, such as trio-binning [26], DipAsm [27] and Hifiasm [28], has facilitated the production of haplotype-resolved genomes.

In the present study, we adopted a trio-binning strategy to build two chromosome-level haplotype-resolved genomes for two *Crassostrea* oyster congeners – *C. angulata* and *C. gigas* – and conducted a comparative genomic analysis. To the best of our knowledge, this is the first report of a fully phased mollusc genome and may further benefit research on molecular ecology, evolution, and genetics in molluscs.

Materials and Methods

Sample collection and sequencing

One hybrid full-sib family was produced by mating a male *C. angulata* (hereinafter referred to as 'AN') oyster from Xiamen, China with a female *C. gigas* ('GI') oyster from Qingdao, China. The two parents and a one-year old offspring ('CH1') were sampled and used for sequencing. Genomic DNA was extracted from the mantle tissues using the standard phenol-chloroform method. Library preparation, quality control, and sequencing were performed according to standard protocols. Short paired-end DNA reads from a WGS library with an insert size of 300 bp were produced for AN, GI and CH1 using the Illumina NovaSeq 6000 system. Short paired-end DNA reads of CH1 were produced from a high-throughput chromosome conformation capture (Hi-C) library with an insert size of 500 base pairs (bp) using the Illumina NovaSeq 6000 system. Long DNA reads from a library with an insert size of 15-20 kbp were generated using the PacBio Smart Sequal II platform. The highly accurate consensus sequence (HiFi) reads were obtained using ccs software version 6.0.0 (github.com/PacificBiosciences/ccs). High quality short paired-end DNA reads were obtained using fastp software version 0.21.0 [29] with the parameters of "-q 20 -u 30 -n 0 -e 20".

Genome survey and reads partition

Basic genome features including genome size, heterozygosity rate, and repeat content were estimated by k-mer-based methods using GenomeScope software version 2.0 [30]. The partitioning of CH1 reads by AN- and GI- unique kmers was conducted using K-Mer Counter software version 3.1.1 [31]. Briefly, the kmer database of kmer size of L for AN (KL_A) and GI (KL_B) were made at L=25, 50, 75 and 100 bp by 'kmc' command with parameters of "-t 20 -ci 1 -cs 1000". The unique kmer database (KLU) was obtained via set difference operation, i.e. AN unique kmer database $KLU_A = KL_A - KL_B$, GI unique kmer database $KLU_B = KL_B - KL_A$, by "kmc_tools kmers_subtract" command with parameters of "-ci 30 -cx 300". Then the distribution of KLU_A and KLU_B kmers were counted for each CH1 reads by "kmc_tools intersect" command. For PacBio HiFi reads, at least two KLU supports were required to group a read. On the other hand, only one KLU support was required for short sequencing reads. Reads without any KLU kmers were considered common reads. Finally, common reads and KLU_A-containing reads were grouped as AN reads; whereas common reads and KLU_B-containing reads were grouped as GI reads.

Assembling and assessment

The partitioned PacBio HiFi reads of AN and GI were separately assembled using Hifiasm program version 0.16.1-r375 with default parameters [28]. The assembly errors of the AN and GI contigs were examined and corrected using Inspector program version 1.0.1. Possible contaminants in contigs were detected using the contaminant screening system on the NCBI genome submission website (https://submit.ncbi.nlm.nih.gov/subs/genome/) and then were removed or fixed manually. Next, the Hi-C DNA reads of AN and GI were mapped to the AN and GI cleaned contigs, respectively, using bwa program version 0.7.17-r1188 [32], and the Hi-C contact matrix was constructed using Juicer software version 1.5 [33]. Finally, 3d-dna program version 180922 [34] was used to further detect and correct the assembly errors, infer the order and orientation of each contig, and link them to chromosome-level scaffolds.

The quality of the final assembly was evaluated as follows. (1) Metazoan BUSCO genes. Quality assessment was conducted using BUSCO software version 5.2.2 [35] with default parameters except a stringent e-value of "1e-5" by searching the genome against 954 metazoan single-copy orthologs from metazoa_odb10 (https://busco-data.ezlab.org/v5/data/lineages/). (2) Short DNA reads mapping. Short WGS DNA reads of AN and GI were mapped to the two genomes using Bwa. In addition, short DNA reads of 20 *C. gigas* individuals from Qingdao and 20 *C. angulata* individuals from Ningde (supplementary file 1) were mapped to the two genomes. The percentage of mapped reads, unique mapped reads, breadth coverage at single base depth ≥ 1, and breadth coverage at a single-base depth ≥ 4 were calculated based on the BAM file using Samtools software version 1.9 [36] to roughly assess the representative of the genome. (3) Transcriptome mapping. A total of 18 *C. angulata* transcriptomes and 18 *C. gigas* transcriptomes in NCBI PRJNA516773 were mapped to the two genomes using the Hisat2 program version 2.1.0 [37]. For a comparison, 18 transcriptomes from another *Crassostrea* oyster *C. ariakensis* in NCBI PRJNA513213 were mapped to the two genomes. The basic mapping statistics were summarized to assess the performance of the genome as a reference for RNA sequencing analysis.

Genome annotation

De novo and homology-based transposable elements (TEs) or interspersed repeats prediction were conducted using RepeatModeler software version 2.0.3 [38] and RepeatMasker software version 4.1.2 [39] respectively. Tandem Repeats Finder (TRF) software version 4.09 [40] was used to detect simple or tandem repeats. The TE-masked genome was used for gene model prediction using homolog-based and RNA-seq-based approaches as described in our previous study [6,10].

Functional annotation of the predicted genes was conducted by means of 5 widely used datasets. NCBI ‘non-redundant’ (NR), Swiss-Prot, and KEGG annotations were retrieved by aligning the proteins to the corresponding database using BlastP software [41] with an E-value threshold of 1e-5; the best hit was retained. Protein domain annotation was executed by searching the InterPro database (https://www.ebi.ac.uk/interpro/) using InterProScan program version 5.34-73.0 (ftp://ftp.ebi.ac.uk/pub/software/unix/iprscan/5/). Gene Ontology (GO) annotations for each gene were obtained by mapping the InterPro entries to GO terms according to the ‘interpro2go’ file.

Comparative genomics and evolutionary analysis

To assess the global similarity of the *C. angulata* and the *C. gigas* genomes, a direct

DNA sequence comparison between the 10 largest scaffolds (pseudo-chromosome sequence 1-10) of AN and GI genome assembly was conducted using the minimap2 program version 2.15-r905 with the parameters of " -t 10 -c -N 2 -Y --eqx -x asm20" [42]. The corresponding fragments whose aligned-region size between two homologous pseudo-chromosome scaffolds were greater than 1000 bp were retained and their sequence similarity was measured by the BLAST identity (gap-uncompressed method, defined as the proportion of identical bases in the full length of the alignments and the gap-compressed identity (gap-compressed method, where consecutive gaps are counted as one gap) (detailed at http://lh3.github.io/2018/11/25/on-the-definition-of-sequence-identity). The sequence divergence rate was calculated by subtracting the sequence identity.

The *C. angulata* and *C. gigas* gene sets, together with gene sets of 13 other mollusc species (11 bivalves and 2 gastropods) and one annelid species from public database (Supplementary Table S1) were collected and ortholog groups were constructed using OrthoFinder software version 2.3.12 [43]. For *C. angulata* and *C. gigas* coding gene comparison, ortholog gene pairs were extracted from the *C. angulata*-*C. gigas* orthogroups. Genes with the same number of CDS were considered to have the same gene model structures. The predicted peptide sequences, coding sequences, intron sequences, and upstream and downstream sequences of the orthologous gene pairs were aligned using Muscle software version 3.8.1551 [44], and the sequence identities were measured by BLAST identity as mentioned above.

To deduce the divergence time of *C. angulata* and *C. gigas*, the single-copy orthologous genes shared by the 16 genomes (Supplementary Table S1) were aligned using Muscle software version 3.8.1551 [44] and then concatenated to construct a maximum-likelihood phylogenetic tree using the IQ-TREE program version 2.2.0 with the parameters of '-m MFP -T 40 -B 10000 --alrt 10000 -bnni' [45]. The divergence time of species was estimated using the MCMCTree program in PAML package version 4.7a [46]. Reference divergence time values (*C. gigas* - *C. virginica*: 63-83 MYA; *E. chlorotica* - *P. canaliculata*: 343-478 MYA) retrieved from the TimeTree database [47] were used to calibrate divergence times on the phylogenetic tree.

To infer the selective pressure of the coding genes, the protein sequences of the ortholog gene pairs between *C. angulata* and *C. gigas* were aligned using Muscle software and based on the amino acid alignments the nucleotide codon alignments were retrieved using the PAL2NAL program [48]. The non-synonymous substitution rate (Ka or d_N), synonymous substitution rate (Ks or d_S) and the ratio of nonsynonymous to synonymous substitution rates (Ka/Ks or d_N/d_S) were calculated using KaKs_Calculator 2.0 with the 'NG' methods [49]. The Ka/Ks values were considered to be statistically significant with a $P < 0.05$ evaluated with Fisher’s Exact test.

For comparative gene family analysis of the 16 genomes, the protein sequences of the predicted coding genes of each genome were aligned to the conserved domain or family profiles in PFAM database [50] using the HMMER program version 3.3.2 [51] with a E-value cutoff of 1e-5. Proteins sharing the same domains were clustered into a single gene family and the gene numbers (GN) in each species was determined. We defined the species expanded gene family using the following criteria: 1) The species with the largest GN. 2) The ratio of maximum GN to second maximum GN was greater than 1.2. 3) The difference between the maximum GN and the second maximum GN was above 3. 4) The ratio of the maximum GN to the average GN of the other species was above 1.5.

A total of 47 wild *C. angulata* oysters collected in Xiamen, China in October, 2022 were resequenced with a raw base production of 15-20 G in the study. Together with 22 *C. angulata* samples from Ningde, and 33, 20, and 33 *C. gigas* oysters from Qingdao, Yantai, and Jinzhou, respectively, produced in our previous study [52], a collection of 69 *C. angulata* and 86 *C. gigas* oysters (supplementary file 1) was used for the resequencing analysis. Briefly, the high-quality resequencing reads were extracted for each individual using fastp software, then mapped to the *C. gigas* genome using bwa program. Subsequently, the bam files were sorted, duplications-removed and indexed using samtools program and SNPs were called using GATK program version 4.1.9.0 [53] with parameters of "QD<2 || FS >60 || MQ < 40 || MQRankSum<-8 || ReadPosRankSum <-8". A phylogenetic tree was constructed using FastME program version 2.0 [54] and the population structure was infered using ADMIXTURE program version 1.3.0 [55]. The nucleotide diversity (θ_π) and wright's fixation index (F_{ST}) were estimated using VCFtools program version 0.1.16 [56]. Linkage disequilibrium decay was analyzed using PopLDdecay program version 3.42 [57]. A SNP was considered species-enriched if the absolute value of the allele frequency difference between the two populations was greater than 0.75 and was considered species-specific if the value was greater than 0.95.

Results

Genome sequencing

A total of 383 G DNA sequencing bases were produced, including 88 G (roughly 147×) bases from 5.49 M highly accurate long DNA reads (HiFi reads) produced by PacBio circular consensus sequencing technology and 295 G Illumina short DNA reads (Table 1). The average length of HiFi reads reached 16 kbp, the average phred-scaled base quality (BQ) was 29.9, and the Q20 and Q30 base percentages were comparable to those of Illumina short DNA reads. During consensus sequencing calling, the average phred-scaled read quality (RQ) and the pass number (PN) were highly positively correlated (Pearson correlation = 0.997, P<1.0e-10); when the PN threshold were 3, 4 and 5, the mean RQs were 29.9, 30.1 and 30.7, respectively.

Table 1 Sequencing data summary.

	AN	GI	CH1		
Species	*C. angulata* (♂)	*C. gigas* (♀)	*C. angulata* × *C. gigas* offspring		
Read Type	Short DNA	Short DNA	Short DNA	Hi-C DNA	PacBio HiFi
Raw Seq (M)	407.80	430.63	470.65	804.98	97.94
Raw Bases (G)	61.17	64.59	70.60	120.75	1404.32
Filtered Seq (M)	366.74	383.34	446.80	770.32	5.49
Filtered Bases (G)	55.01	57.50	67.02	115.55	88.01
Filtered Q20 (%)	97.86	98.01	97.66	98.28	98.13
Filtered Q30 (%)	93.40	93.83	93.11	94.39	95.71
Average size (bp)	150	150	150	150	16032
Coverage (×)	92	96	112	193	147

Genome survey and reads partition

The estimated genome size, heterozygosity rate, and repeat content of AN, GI and CH1 were 572.3 M (2.6%, 41.6%), 594.4 M (2.9%, 43.7%) and 579.4 M (3.3%, 42.3%), respectively (Fig. 1a-c). As expected, the genome size and repeat content of CH1 were both approximately the average of those of the two parents; whereas the CH1 heterozygosity rate was significantly higher than that of AN and GI.

A large number of unique kmers were found in the AN and GI WGS DNA reads (Fig. 1d). For AN, at kmer sizes of 25, 50, 75 and 100 bp, there were approximately 590, 670, 538 and 246 M kmers, respectively; and the corresponding unique kmer numbers were 110, 124, 79 and 29 M respectively. For GI, there were approximately 614, 725, 616 and 279 M kmers; and the unique kmer numbers were 99, 118, 69 and 23 M, respectively. Although the total number of GI kmers was slightly higher than that of AN, the number of unique kmers of GI was lower than that of AN. In AN and GI, the unique kmers accounted for 11.7-18.6% and 8.2-16,1%, respectively, of the total kmers.

All types of CH1 sequencing reads were grouped using AN- and GI-unique kmers to constitute AN- and GI-originated read sets (Fig. 1e). More than 97% of the HiFi reads can be effectively partitioned, including 48.9% of AN reads, 48.7% of GI and 0.6% of common reads. For the Hi-C short DNA reads, the AN, GI, and common reads accounted for 36%, 35%, and 25%, respectively, of the total reads. Similarly, for the WGS short DNA reads, the AN, GI, and common reads accounted for 35%, 34%, and 31%, respectively, of the total reads.

Genome assembly and assessment

Portioned HiFi reads of CH1 were used to independently construct contigs with high contiguity for the two parents (AN and GI) to generate two fully haplotype-resolved or phased genome assemblies. Using the portioned Hi-C reads of CH1, the 3D proximities of the contig pairs in each phased genome were deduced from the Hi-C contact matrix, and most contigs were well placed in the scaffolding process (Fig. 2a-b). The assembly size and contig N50 of the AN genome were 582.3 M and 12.7M, respectively; and those of the GI genome were 606.3 M and 5.5M, respectively (Table 2; Fig. 2c).

Table 2 Assembly statistics of the two haplotype-resolved genomes.

	C. angulata		*C. gigas*	
	contig	scaffold	contig	scaffold
Sequence Number	166	75	293	88
Assembly Size (M)	582.28		606.27	
Longest SeqLen (M)	22.47	70.14	25.30	84.89
Shortest SeqLen (K)	13.94	14.50	16.33	19.02
Average SeqLen (M)	3.51	7.76	2.07	6.89
N50 (M)	12.78	60.09	5.46	60.54
L50	18	5	35	5
N95 (M)	1.63	35.28	0.83	36.64
L95	67	10	139	10

The scaffold L95 of the two genomes was equal to 10, which is the expected haploid number of the *C. gigas* genome. This suggests that in each genome, the 10 pseudo-chromosome sequences consisting of the 10 longest scaffolds may well represent the overwhelming majority of the whole-genome contents. Similar to the kmer-based genome size estimation, the assembly size of AN was approximately 24 M smaller than that of GI. On the other hand, with the exception of pseudo-chromosome 1, the size differences of the remaining corresponding pseudo-chromosome sequences of the two genomes were much smaller (Fig. 2d).

BUSCO assessment using 954 metazoan single-copy orthologs revealed that the proportion of complete (C), complete and single-copy (S), completely duplicated (D), fragmented (F), and missing (M) genes of the *C. angulata* genome and the *C. gigas* genome was [C:99.1%; S:98.3%; D:0.8%; F:0.6%; M:0.3%] and [C:98.9%; S:98.0%; D:0.9%; F:0.5%; M:0.6%], respectively, implying the improved assembly quality in comparison with the several previously published *Crassostrea* oyster genomes (Fig. 2c; supplementary file 2).

Using *C. angulata* genome as a reference, the overall mapping rates of WGS short reads of AN, CH1, and GI were 98.36%, 98.21%, and 97.25%, respectively, showing a very slight gradual decreasing trend. Using *C. gigas* genome as a reference, the overall mapping rates of WGS short reads of GI, CH1, and AN were 98.54%, 98.30%, and 97.18%, respectively, showing the same trend observed above (Supplementary Table S2). At the population level, the average mapping rates of WGS short reads of 20 *C. angulata* and 20 *C. gigas* oysters using *C. angulata* as the reference were 94.16 ± 0.19% and 93.47 ± 0.45%, respectively. The average mapping rates were 93.67 ± 0.21% and 94.00 ± 0.41% when using *C. gigas* as the reference.

The mean mapping rates when mapping the transcriptome reads of *C. angulata* and *C. gigas* to the two genomes were between 73 and 77%. However, 3 to 4% differences were observed when mapping to their own genome in comparison with mapping to another genome. A similar trend was observed for unique mapping rates. In contrast, the map rate of mapping the transcriptome reads of the distantly related oyster, *C. ariakensis* to the two genomes was both less than 24%, and the map rate of mapping the *C. angulata* and *C. gigas* transcriptome reads to *C. ariakensis* genome was both less than 18%. (Supplementary Table S2).

Repeat sequences and gene annotation

By combining repeat detection using *de novo* and homology-based methods, a total of 279.7 M of repetitive sequences were identified in the *C. angulata* genome, accounting for 48.0% of the genome. The repeat contents of the two genomes were nearly identical at the whole-genome level and between the 10 pairs of pseudo-chromosome sequences (Supplementary Table S3). In both genomes, interspersed repeats dominated and the tandem repeat percentages were less than 5%. The overall repeat content was at a medium level in the animal kingdom and was comparable to that of other *Crassostrea* genomes.

A total of 28,211 and 28,441 coding genes were predicted in the two genomes, and more than 21,584 (76.5%) and 21,740 (76.4) coding genes could be annotated using at least two types of publicly protein datasets (Table 3). The two gene sets were highly similar in terms of gene/CDS number, gene/CDS length and percentage, number of

genes with different exon numbers, and number of genes with different annotations. Most of the genes (79–80%) contained 2–20 exons, with single exon genes accounting for approximately 14% and less than 7% of the genes containing more than 20 exons.

Table 3 Gene prediction and annotation summary of the two genomes

	C. angulata	*C. gigas*
Gene no.	28,211	28,441
Total CDS len. (M)	42.91 (7.37%)	43.01 (7.10%)
Mean CDS len.	1,521	1,512
Total gene len. (M)	209.01 (35.90%)	206.48 M (34.06%)
Mean gene len.	7,409	7,259
CDS =1	3,852 (13.65%)	3,963 (13.93%)
CDS 2-10	17,940 (63.59%)	18,094 (63.62%)
CDS 11-20	4,522 (16.03%)	4,522 (15.90%)
CDS >20	1,897 (6.72%)	1,862 (6.54%)
NR	27,544 (97.64%)	27,696 (97.38%)
Swiss-Prot	14,824 (52.55%)	14,763 (51.91%)
KEGG	10,210 (36.19%)	10,193 (35.84%)
InterPro	20,892 (74.06%)	20,978 (73.76%)
GO	13,884 (49.22%)	13,884 (48.82%)
no. of ≥1 anno.	27,589 (97.80%)	27,755 (97.59%)
no. of ≥2 anno.	21,584 (76.51%)	21,740 (76.44%)

Comparative genomics and evolutionary analysis

A direct comparison of the DNA sequences of the two organisms revealed an overall pairwise alignment identity of greater than 0.75 at the whole genome level (Fig. 3a). Although repeats (such as the widespread interspersed repetitive elements) could lead to alignments at multiple positions, similar DNA fragments in the 10 pseudo-chromosomes of *C. angulata* with its counterparts of *C. gigas* constituted most of the larger conserved DNA sequence block pairs. This implies significant synteny and high genomic similarity between the two assemblies (Fig. 3a-b). Detailed parsing of the alignments of conserved segments in the 10 pairs of pseudo-chromosomes found that the BLAST identity medians (means) were 0.85–0.87 (0.72–0.80), whereas the gap-compressed identity medians (means) were 0.95–0.97 (0.96–0.97) (Fig. 3c). The total alignment, match, mismatch and indel sizes were about 357.6 M, 274.7 M, 7.58 M and 75.26 M, respectively (Fig. 3d). Moreover, the average sequence divergence rates calculated by gap-uncompressed and gap-compressed methods were 0.232 and 0.031, respectively. It is obvious that gaps (indels) can cause a much larger number of alignment differences in the calculation of sequence identity (Fig. 3d). The larger indels of ≥ 50 bp (usually considered as one kind of structural variation) had a total size of 51.36 M, accounting for 68.3% of the total indel length. Thus, the divergence rate of the two genomes estimated by

nucleotide substitution (mismatch), small indels (gaps, < 50 bp) and big indels (gaps, ≥50 bp) were 0.021, 0.067, and 0.144, respectively. This indicates that structural variations such as deletions and insertions were the major sources leading to the genomic divergences of *C. angulata* and *C. gigas*.

The construction of orthologous groups of two or more genomes underlies comparative and phylogenetic analyses of gene sets at the coding gene level. A total of 34,043 orthologous groups were identified in *C. angulata* and *C. gigas* together with 14 other genomes. From the comparison between the two genomes, the number of orthologous genes in the four subtypes (i.e., one-to-one, one-to-many, many-to-one, and many-to-many orthologs) were 21055, 1080, 2427, 484, and 21055, 1013, 2579, and 477 in the *C. angulata* and *C. gigas* genomes, respectively (Fig. 4a). Most of these orthologs were located in the corresponding pseudo-chromosome pairs in the two genomes, and the genomic position orders of the one-to-one orthologs were strongly correlated (Spearman's rank correlation rho = 0.966, P < 1.0e-10). This suggests a distribution pattern characterized by highly conserved spatial collinearity (Fig. 4b). The level of sequence conservation varied dramatically across different gene regions. The average coding sequences ('cds'), deduced protein sequences ('pep'), introns ('int'), up-stream 10Kbp segments ('up10k') and down-stream 10Kbp segments ('dn10k') identities of the orthologous gene pairs of the two genomes were 0.8894, 0.8776, 0.6156, 0.6032 and 0.6215, respectively. The average sequence identities of the upstream and downstream regions gradually decreased with increasing distance from the CDS (Mann-Kendall trend test, P < 1.0e-4) (Fig. 4c). Gene structure (referring to the number of CDS) had an impact on sequence identities. The average 'cds' ('pep', 'int', 'up10k', 'dn10k') identities of 21012 gene pairs with identical gene structure were 0.9479 (0.9405, 0.6881, 0.6371, 0.6516) and were significantly higher than those of 5847 gene pairs ((0.6791 (0.6517, 0.3554, 0.4811, 0.5135)) with different gene structure (Welch Two Sample t-test, P < 1.0e-10). There were a total of 15475 highly conserved ortholog pairs, which shared identical gene structure and both the 'cds' and the 'pep' identities were bigger than 0.90. A further investigation on the alignments of the 15475 gene pairs revealed that the indels located in the intron, 'up-2k' and 'dn2k' were the major elements leading to the sequence divergence of orthologous gene regions. The number of ortholog pairs that harbored indels with size of > 10 bp in the CDS, intron, 'up-2k' and 'dn2k' regions were 1862, 13181, 13304 and 12742, respectively (Fig. 4d). The number of ortholog pairs that harbored indels with size of >50bp in the CDS, intron, 'up-2k' and 'dn2k' regions were 471, 10565, 6431, and 5756, respectively.

Based on the orthologous gene inference of multiple species, 519 single-copy genes were identified and submitted for the construction of a species phylogenetic tree. *C. angulata* and *C. gigas* were first clustered into a clade, and their divergence time was estimated to be 4.82 MYA [95% confidence interval (3.31, 6.76)] (Fig. 5a). This was far shorter than the divergence times among other *Crassostrea* species. Ka/Ks analysis of the orthologs of *C. angulata* and *C. gigas* revealed that the vast majority of Ka and Ks values were less than 0.1, most of the Ka/Ks values were below 0.4, and only 17 gene pairs had Ka/Ks values greater than 1 (Fig. 5b). Gene annotations showed that

only six of the 17 genes had SWISS-PROT matches, and that the NR matches of the remaining 11 genes were mostly uncharacterized proteins (Supplementary Table S4). The average transcript TPM values of 11 of the 18 genes were greater than 2.0 in the gills under normal physiological conditions. Gene family domain analysis revealed 21 expanded gene families, of which 9 were in *C. angulata*, another 9 were in *C. gigas*, and 3 were in both species (Fig.5c; Supplementary Table S5). In *C. angulata*, the 'Histone'-related gene families were significantly expanded: there were 127 genes with 'Histone' domain (PF00125: Core histone H2A/H2B/H3/H4) and 47 genes with 'Linker_histone' domain (PF00538: linker histone H1 and H5 family). The maximum GNs of the two gene families in other species were 85 and 29, respectively. *C. angulata* had 22 genes with 'Carboxyl_trans' domain (PF01039: Carboxyl transferase domain), whereas *C. gigas* had 10 and other species only had 3 to 9. In *C. gigas*, the GN of 'zf-H2C2' (PF09337: H2C2 zinc finger) family was 63, which was much higher than the GN of 40 in *C. angulata* and 0 to 23 in other species. The 'H_lectin' (PF09458: H-type lectin domain), 'SCAN' (PF02023: SCAN domain) and 'KDZ' (PF18758: Kyakuja-Dileera-Zisupton transposase) families were also enriched in *C. gigas*. Only three families (PF17917: RNase H-like domain found in reverse transcriptase; PF01608.18: I/LWEQ domain; and PF06021: Aralkyl acyl-CoA:amino acid N-acyltransferase) were overrepresented in both organisms.

Based on the resequencing data from 69 *C. angulata* and 86 *C. gigas* oysters, a total of 15.1 M high-confidence bi-allelic SNPs with MAF > 0.01 and missing rate < 0.05 were identified. The two species were clearly clustered into two large groups based on phylogenetic analysis and population structure inference (Fig. 6a-b). Within each species, genetic admixing was observed in some individuals from different locations and the oysters could not be fully separated based on their sampling locations. At the whole genome level, the nucleotide diversity (θ_π) of *C. gigas* and *C. angulata* were 4.13×10^{-3} and 3.94×10^{-3} respectively, and the former was slightly higher than the latter. At each of the 10 pseudo-chromosomes, the θ_π of *C. gigas* was also slightly higher than that of *C. angulata* (Kolmogorov-Smirnov test, $P < 0.05$) (Fig. 6c).. Rapid linkage disequilibrium decay was observed for both species, and the r^2 values decreased from 0.3 to 0.15 within a 200 to 300 bp span (Fig. 6d).

For 93.1% of the SNPs, the allele frequency difference between the two populations was less than 0.30. A total of 82,245 species-enriched and 2,756 species-specific SNPs had allele frequency differences greater than 0.75 and 0.95. The proportion of species-enriched 'intergenic-', 'downstream-', 'upstream-', 'intron-', 'synonymous-', and 'nonsynonymous-' SNPs in the total SNPs of the above 6 types were 0.007474, 0.007022, 0.006609, 0.007196, 0.006716, and 0.008383, respectively. The proportions of species-specific SNPs among the above six types were 0.000265, 0.000207, 0.000244, 0.000218, 0.000188, and 0.000325, respectively. In both cases, the proportion of nonsynonymous SNPs was significantly higher than that of other types of SNPs (chi-square tests, $P < 0.05$).

The *F*st median of the two populations estimated by the 10 kbp sliding windows was 0.055. Additionally, the genomic regions with the largest *F*st (> 0.152, top 5%) and θ_π ratio divergence (< 0.70 or > 1.96; bottom and top 5%) was around 13.2 Mbp, overlapping 1,088 coding genes (Fig. 6e). In total, 704 and 384 putative genes under

selection were identified in *C. angulata* and *C. gigas*, respectively (Supplementary Table S6). In *C. angulata*, these genes were enriched in 25 pathways, including the cGMP-PKG signaling pathway, pentose phosphate pathway, fat digestion and absorption, protein digestion and absorption, and the HIF-1 signaling pathway. In *C. gigas,* genes were enriched in seven pathways, including protein digestion and absorption, ovarian steroidogenesis, and progesterone-mediated oocyte maturation (Supplementary Table S7). In addition, selection signals were detected in two heat shock 70 kDa protein (HSP70) genes and one HSP90 gene in *C. angulata* and *C. gigas*.

Discussion

Because of their considerable roles in aquatic ecological systems and as food or industrial materials for humans, mollusks have attracted more research attention than ever, and high-quality genomes have gradually become a necessary resource for basic research. It has become common for distinct research groups to publish genome assemblies for the same species or release several genomes simultaneously for different species [9,10,15,16,18,19,58]. Multiple genomes of the same organism once seemed unnecessary when sequencing was too expensive and a standard reference was sufficient for most analyses. However, they are now considered essential in an era when costs are dramatically reduced and more focus is paid to the exploration of different levels of genomic variations in the scenario of a pan-genome framework [59]. Here, we provided genomes for two closely related *Crassostrea* oyster congeners, the Portuguese oyster *C. angulata* and the Pacific oyster *C. gigas*. We performed comparative studies at the single-genome and population levels, which presented improved assembly qualities and may further deepen our understanding of oyster genome diversities.

Oysters and other bivalves have high levels of genomic polymorphisms [5,60], which are the main barriers to a high-quality assembly. A traditional method to reduce heterozygosity is inbreeding, but it is quite difficult to obtain individuals with high inbreeding coefficient and maintain multiple-generation inbreeding strains in bivalves; this strategy has been applied to genome projects only in a few species, such as the Pacific oyster [6] and Yesso scallop [61]. Even assisted by the fosmid-pooling hierarchical assembly approach, the first version of the Pacific oyster genome was fragmented, with a contig N50 of several kilobases and a scaffold N50 of several hundred kilobases, which was of the same order of magnitude as the later-appearing genomes of other bivalves produced by similar sequencing strategies. High heterozygosity and repetitive sequences can result in redundancy and imperceptible assembly errors in contigs [62,63]. Long DNA reads spanning repeats are key to maximizing genome quality. Based on long DNA reads and HiC scaffolding, nearly all bivalves genomes released in the past 2-3 years were at the chromosome level with contig N50 >1 million bases. BUSCO evaluation indicated that several of the previously published *Crassostrea* oyster genomes had higher completeness (C >95%) and however, none of them had a result of 'S >95%, D <1%, F <1%, M <1%', and most of the duplicated BUSCOs were >2.5% (supplementary file 2), implying possible redundancies. In the present study, BUSCOs of the two genomes reached 'S >98%, D <1%, F <1%, M <1%' and contig N50 were both >5.0 M (*C. angulata* >12M), demonstrating an significant improvement in basic assembly quality assessment metrics.

The *C. angulata* and *C. gigas* genomes were chromosome-level and fully haplotype-resolved, which is the most typical feature of these two genomes. To date, complete phased genomes have only been accomplished in several species [20,23,24], although the trend for building phased genomes and their advantages in related studies has been widely accepted. A major challenge is the lack of global phase information for separating haplotypes over long genomic distances [24]. The trio-binning strategy can group the sequencing reads of a diploid genome by leveraging parent-specific k-mers, thus simplifying the haplotype assembly [26]. A basic requirement for read binning is the isolation of an adequate number of parent-specific K-mers [64]. In this study, a high proportion of unique kmers of four lengths could effectively separate more than 97% of the CH1 long reads and 61–91% of the short DNA reads of Hi-C and WGS. This suggests that trio binning could turn the high-heterozygosity disadvantage that once hindered genome assembly into a distinct advantage. The present study demonstrated that the trio-binning strategy is an effective approach for building haplotype-derived bivalve genomes.

The two genomes had similar features in terms of GC content, repeat content, coding gene numbers and sizes, and gene annotations, which were comparable to those of other *Crassostrea* genomes. However, their heterozygosity was significantly higher than that of the Jinjiang and Hong Kong oysters, as estimated either by kmer or resequencing analysis [16,17]. A direct comparison of the two genomes revealed large number of conserved DNA sequence block pairs and an average gap-compressed identity of greater than 0.96, implying high similarity and significant synteny of the two genomes. Many studies have used the *C. gigas* genome as a reference to align *C. angulata* sequencing reads for subsequent analysis [52,65]. Considering the read mapping rate, the difference was negligible. In contrast, large insertions and deletions were common between the two genomes and could lead to a divergence rate of 0.144, indicating that structural variations were the major elements that varied between the two genomes. Structural variations are an important source of genetic diversity [66], and many copy number variations have been reported in the Pacific oyster *C. gigas* and the eastern oyster *C. virginica* genomes [10,67,68].

In the genus *Crassostrea*, the divergence time of *C. angulata* and *C. gigas* was 4.82 MYA, even though they were the most closely related species. This is greater than the 2.72 MYA estimated by mitochondrial genes [69]. This may be because 209 single-copy genes were used in the present study, whereas only 12 coding genes were used in the previous study. A total of 21,055 one-to-one ortholog gene pairs were identified, and 15,475 shared identical gene structures, > 90% identity in coding and protein sequences, and highly conserved spatial collinearity. This could largely explain the previous report that found high macro-collinearity and the same order of most of the transferable EST markers in *C. angulata* and *C. gigas* genomes [70]. The low Ks value and Ka/Ks ratios suggested that most of the orthologous genes were conserved between the two genomes and were subject to strong selective constraints [49].

C. gigas and *C. angulat*a have similar external morphological features, hybridize under natural conditions, and produce fertile offspring [3,71,72]. Previous studies have suggested that *C. angulata* was a subspecies of *C. gigas* [1]. The significant synteny and high genomic similarity of the two assemblies, the large number of highly conserved ortholog gene pairs, and population analysis in this study could provide novel evidences supporting this view. Although both species had large intra- and inter-individual polymorphisms, *Fst* estimation indicated that most of the genomic regions showed low to moderate levels of genetic differentiation, which is in

accordance with our previous report [65]. An increasing number of studies have found physiological differences between the two species in terms of growth, thermal tolerance, fatty acid content and composition [3,12,73], as well as adaptive divergence of plasticity in environmentally responsive genes [74]. In the present study, 1,088 coding genes were identified as candidate genes possibly under selection. These genes included HSP70 and HSP90, which are key molecules in protein homeostasis, thermal adaptation, and stress response [75]. Genes related to fat and protein digestion and absorption were enriched, which was in accordance with our previous work showing that energy metabolism plays a considerable role in the formation of adaptive traits in the two species [12,13]. These genes could provide new resources for understanding the evolution and connections between genes and biological features of the two congeneric oyster species.

Conclusion

Two chromosome-level fully phased genomes were constructed for the Portuguese oyster *C. angulata* and the Pacific oyster *C. gigas* through a trio-binning strategy. They were characterized by high BUSCO completeness and contig N50 size and ranked at the top of marine invertebrate genomes with high contiguity and integrity. The general features of the two genomes were similar and 15,475 highly conserved orthologous gene pairs were identified. At the population level, individuals of the two species were clearly clustered into two large groups; 2,756 species-specific SNPs and 1,088 coding genes, possibly under selection, were identified. The study provides novel data resources that contribute to the genomics, genetics and evolution studies in molluscs.

Acknowledgements

We thank the staff in the high performance computing center (HPCC) of the Institute of Oceanology for their assistance with bioinformatics software installation. We thank Dr. Ximing Guo for suggestions on sequencing strategy.

Competing Interests

The authors declare that they have no competing interests.

Funding

The work was supported by the National Key R&D Program of China (2022YFD2400301), the National Natural Science Foundation of China (41876169), the Key Research and Development Program of Shandong (2022LZGC015), and the Earmarked Fund for China Agriculture Research System (No. CARS-49).

Data Availability

The genomes and raw sequencing reads produced in the study have been released in the NCBI database. Genome assemblies: *C. angulata*, GCA_025765675.2; *C. gigas*, GCA_025765685.2; Sequencing reads: paternal *C. angulata*, SRR21185640; maternal *C. gigas*, SRR21185639; the hybrid offspring, SRR21185636, SRR21185637, SRR21185638; 47 newly resequenced *C. angulata* oysters: SRR22668975 - SRR22669021. All of the sequencing data used in the study are detailed in supplementary file 1.

Authors' Contributions

HQ: project design, data analysis and paper writing. RC: oyster family construction and culture. YW: sequencing data processing and management. LL and GZ: project organization and funding supports.

Supplementary Files

Supplementary file 1: Notes for the usage of resequenced data.
Supplementary file 2: The BUSCOs of the several *Crassostrea* oyster genomes.
Supplementary Table S1: The genomes used for ortholog group construction.
Supplementary Table S2: Genome assessment by short DNA reads and transcriptome mapping.
Supplementary Table S3: The repeat contents of the two genomes.
Supplementary Table S4: The gene pairs with Ka/Ks > 1.
Supplementary Table S5: The expanded gene families defined by PFAM domains.
Supplementary Table S6: The putative genes under selection in C. *angulata* and C. *gigas*.
Supplementary Table S7: The enriched KEGG pathways of the genes under selection.

References

1. Wang HY, Qian LM, Liu XA, et al. Classification of a Common Cupped Oyster from Southern China. J Shellfish Res. 2010;29(4):857-66.
2. Foighil DO, Gaffney PM, Wilbur AE, et al. Mitochondrial cytochrome oxidase I gene sequences support an Asian origin for the Portuguese oyster Crassostrea angulata. Mar Biol. 1998;131(3):497-503.
3. Huvet A, Gerard A, Ledu C, et al. Is fertility of hybrids enough to conclude that the two oysters Crassostrea gigas and Crassostrea angulata are the same species? Aquat Living Resour. 2002;15(1):45-52.
4. Reece KS, Cordes JF, Stubbs JB, et al. Molecular phylogenies help resolve taxonomic confusion with Asian Crassostrea oyster species. Mar Biol. 2008;153(4):709-21.
5. Hedgecock D, Gaffney PM, Goulletquer P, et al. The case for sequencing the Pacific oyster genome. J Shellfish Res. 2005;24:429-41.
6. Zhang G, Fang X, Guo X, et al. The oyster genome reveals stress adaptation and complexity of shell formation. Nature. 2012;490(7418):49-54.
7. Hedgecock D, Shin G, Gracey AY, et al. Second-Generation Linkage Maps for the Pacific Oyster Crassostrea gigas Reveal Errors in Assembly of Genome Scaffolds. G3-Genes Genom Genet. 2015;5(10):2007-19.
8. Picot S, Faury N, Arzul I, et al. Identification of the autophagy pathway in a mollusk bivalve, Crassostrea gigas. Autophagy. 2020;16(11):2017-35.
9. Penaloza C, Gutierrez AP, Eory L, et al. A chromosome-level genome assembly for the Pacific oyster Crassostrea gigas. Gigascience. 2021;10(3).
10. Qi H, Li L, Zhang G. Construction of a chromosome-level genome and variation map for the Pacific oyster Crassostrea gigas. Mol Ecol Resour. 2021;21(5):1670-85.
11. Gagnaire PA, Lamy JB, Cornette F, et al. Analysis of Genome-Wide Differentiation between Native and Introduced Populations of the Cupped Oysters Crassostrea gigas and Crassostrea angulata. Genome Biol Evol. 2018;10(9):2518-34.
12. Wang CG, Li A, Wang W, et al. Integrated Application of Transcriptomics and Metabolomics Reveals the Energy Allocation-Mediated Mechanisms of Growth-Defense Trade-Offs in Crassostrea gigas and Crassostrea angulata. Front Mar Sci. 2021;8.
13. Li A, Li L, Song K, et al. Temperature, energy metabolism, and adaptive divergence in two oyster subspecies. Ecol Evol. 2017;7(16):6151-62.
14. Gomes-dos-Santos A, Lopes-Lima M, Castro LFC, et al. Molluscan genomics: the road so far and the way forward. Hydrobiologia. 2020;847(7):1705-26.
15. Wu B, Chen X, Yu MJ, et al. Chromosome-level genome and population genomic analysis provide insights into the evolution and environmental adaptation of Jinjiang oyster Crassostrea ariakensis. Mol Ecol Resour. 2022;22(4):1529-44.
16. Li A, Dai H, Guo XM, et al. Genome of the estuarine oyster provides insights into climate impact and adaptive plasticity. Commun Biol. 2021;4(1).
17. Zhang Y, Mao F, Xiao S, et al. Comparative Genomics Reveals Evolutionary Drivers of Sessile Life and Left-right Shell Asymmetry in Bivalves. Genom Proteom Bioinf. 2022.
18. Boutet I, Monteiro HJA, Baudry L, et al. Chromosomal assembly of the flat oyster (Ostrea edulis L.) genome as a new genetic resource for aquaculture. Evol Appl. 2022;15(11):1730-48.

19. Gundappa MK, Penaloza C, Regan T, et al. Chromosome-level reference genome for European flat oyster (Ostrea edulis L.). Evol Appl. 2022;15(11):1713-29.
20. Low WY, Tearle R, Liu RJ, et al. Haplotype-resolved genomes provide insights into structural variation and gene content in Angus and Brahman cattle. Nat Commun. 2020;11(1).
21. Ebert P, Audano PA, Zhu Q, et al. Haplotype-resolved diverse human genomes and integrated analysis of structural variation. Science. 2021;372(6537).
22. Zhang XT, Wu RX, Wang YB, et al. Unzipping haplotypes in diploid and polyploid genomes. Comput Struct Biotec. 2020;18:66-72.
23. Zhou Q, Tang D, Huang W, et al. Haplotype-resolved genome analyses of a heterozygous diploid potato. Nat Genet. 2020;52(10):1018-23.
24. Porubsky D, Ebert P, Audano PA, et al. Fully phased human genome assembly without parental data using single-cell strand sequencing and long reads. Nat Biotechnol. 2021;39(3):302-8.
25. Holley G, Beyter D, Ingimundardottir H, et al. Ratatosk: hybrid error correction of long reads enables accurate variant calling and assembly. Genome Biol. 2021;22(1).
26. Koren S, Rhie A, Walenz BP, et al. De novo assembly of haplotype-resolved genomes with trio binning. Nat Biotechnol. 2018;36(12):1174-82.
27. Garg S, Fungtammasan A, Carroll A, et al. Chromosome-scale, haplotype-resolved assembly of human genomes. Nat Biotechnol. 2021;39(3):309-12.
28. Cheng HY, Concepcion GT, Feng XW, et al. Haplotype-resolved de novo assembly using phased assembly graphs with hifiasm. Nat Methods. 2021;18(2):170-5.
29. Chen S, Zhou Y, Chen Y, et al. fastp: an ultra-fast all-in-one FASTQ preprocessor. Bioinformatics. 2018;34(17):i884-i90.
30. Vurture GW, Sedlazeck FJ, Nattestad M, et al. GenomeScope: fast reference-free genome profiling from short reads. Bioinformatics. 2017;33(14):2202-4.
31. Kokot M, Dlugosz M, Deorowicz S. KMC 3: counting and manipulating k-mer statistics. Bioinformatics. 2017;33(17):2759-61.
32. Li H, Durbin R. Fast and accurate short read alignment with Burrows-Wheeler transform. Bioinformatics. 2009;25(14):1754-60.
33. Durand NC, Shamim MS, Machol I, et al. Juicer Provides a One-Click System for Analyzing Loop-Resolution Hi-C Experiments. Cell Syst. 2016;3(1):95-8.
34. Dudchenko O, Batra SS, Omer AD, et al. De novo assembly of the Aedes aegypti genome using Hi-C yields chromosome-length scaffolds. Science. 2017;356(6333):92-5.
35. Simao FA, Waterhouse RM, Ioannidis P, et al. BUSCO: assessing genome assembly and annotation completeness with single-copy orthologs. Bioinformatics. 2015;31(19):3210-2.
36. Li H, Handsaker B, Wysoker A, et al. The Sequence Alignment/Map format and SAMtools. Bioinformatics. 2009;25(16):2078-9.
37. Kim D, Landmead B, Salzberg SL. HISAT: a fast spliced aligner with low memory requirements. Nat Methods. 2015;12(4):357-60.
38. Flynn JM, Hubley R, Goubert C, et al. RepeatModeler2 for automated genomic discovery of transposable element families. P Natl Acad Sci USA. 2020;117(17):9451-7.
39. Tarailo-Graovac M, Chen N. Using RepeatMasker to identify repetitive elements in genomic sequences. Curr Protoc Bioinform. 2009;25:4.10.1-4..4.
40. Benson G. Tandem repeats finder: a program to analyze DNA sequences. Nucleic Acids Res. 1999;27(2):573-80.

41. Altschul SF, Gish W, Miller W, et al. Basic Local Alignment Search Tool. J Mol Biol. 1990;215(3):403-10.
42. Li H. Minimap2: pairwise alignment for nucleotide sequences. Bioinformatics. 2018;34(18):3094-100.
43. Emms DM, Kelly S. OrthoFinder: phylogenetic orthology inference for comparative genomics. Genome Biol. 2019;20(1).
44. Edgar RC. MUSCLE: multiple sequence alignment with high accuracy and high throughput. Nucleic Acids Res. 2004;32(5):1792-7.
45. Minh BQ, Schmidt HA, Chernomor O, et al. IQ-TREE 2: New Models and Efficient Methods for Phylogenetic Inference in the Genomic Era. Mol Biol Evol. 2020;37(5):1530-4.
46. Yang ZH. PAML 4: Phylogenetic analysis by maximum likelihood. Mol Biol Evol. 2007;24(8):1586-91.
47. Kumar S, Stecher G, Suleski M, et al. TimeTree: A Resource for Timelines, Timetrees, and Divergence Times. Mol Biol Evol. 2017;34(7):1812-9.
48. Suyama M, Torrents D, Bork P. PAL2NAL: robust conversion of protein sequence alignments into the corresponding codon alignments. Nucleic Acids Res. 2006;34:W609-W12.
49. Zhang Z. KaKs_Calculator 3.0: Calculating Selective Pressure on Coding and Non-coding Sequences. Genom Proteom Bioinf. 2022;20(3):536-40.
50. Finn RD, Bateman A, Clements J, et al. Pfam: the protein families database. Nucleic Acids Res. 2014;42(D1):D222-D30.
51. Wheeler TJ, Eddy SR. nhmmer: DNA homology search with profile HMMs. Bioinformatics. 2013;29(19):2487-9.
52. Li L, Li A, Song K, et al. Divergence and plasticity shape adaptive potential of the Pacific oyster. Nat Ecol Evol. 2018;2(11):1751-60.
53. McKenna A, Hanna M, Banks E, et al. The Genome Analysis Toolkit: A MapReduce framework for analyzing next-generation DNA sequencing data. Genome Res. 2010;20(9):1297-303.
54. Lefort V, Desper R, Gascuel O. FastME 2.0: A Comprehensive, Accurate, and Fast Distance-Based Phylogeny Inference Program. Mol Biol Evol. 2015;32(10):2798-800.
55. Alexander DH, Novembre J, Lange K. Fast model-based estimation of ancestry in unrelated individuals. Genome Res. 2009;19(9):1655-64.
56. Danecek P, Auton A, Abecasis G, et al. The variant call format and VCFtools. Bioinformatics. 2011;27(15):2156-8.
57. Zhang C, Dong SS, Xu JY, et al. PopLDdecay: a fast and effective tool for linkage disequilibrium decay analysis based on variant call format files. Bioinformatics. 2019;35(10):1786-8.
58. Bean TP, Tanguy A, Penaloza C, et al. Two parallel chromosome-level reference genomes to support restoration and aquaculture of European flat oyster Ostrea edulis. Evol Appl. 2022;15(11):1709-12.
59. Yang X, Lee WP, Ye K, et al. One reference genome is not enough. Genome Biol. 2019;20(1):104.
60. Takeuchi T. Molluscan Genomics: Implications for Biology and Aquaculture. Current Molecular Biology Reports. 2017;3(4):297-305.
61. Wang S, Zhang JB, Jiao WQ, et al. Scallop genome provides insights into evolution of bilaterian karyotype and development. Nat Ecol Evol 2017;1(5).
62. Guan D, McCarthy SA, Wood J, et al. Identifying and removing haplotypic duplication in primary genome assemblies. Bioinformatics. 2020;36(9):2896-8.

63. Torresen OK, Star B, Mier P, et al. Tandem repeats lead to sequence assembly errors and impose multi-level challenges for genome and protein databases. Nucleic Acids Res. 2019;47(21):10994-1006.
64. Kronenberg ZN, Rhie A, Koren S, et al. Extended haplotype-phasing of long-read de novo genome assemblies using Hi-C. Nat Commun. 2021;12(1).
65. Li A, Li L, Zhang Z, et al. Noncoding Variation and Transcriptional Plasticity Promote Thermal Adaptation in Oysters by Altering Energy Metabolism. Mol Biol Evol. 2021;38(11):5144-55.
66. Escaramis G, Docampo E, Rabionet R. A decade of structural variants: description, history and methods to detect structural variation. Brief Funct Genomics. 2015;14(5):305-14.
67. Jiao ZX, Tian Y, Hu BY, et al. Genome Structural Variation Landscape and Its Selection Signatures in the Fast-growing Strains of the Pacific Oyster, Crassostrea gigas. Mar Biotechnol. 2021;23(5):736-48.
68. Modak TH, Literman R, Puritz JB, et al. Extensive genome-wide duplications in the eastern oyster (Crassostrea virginica). Philos T R Soc B. 2021;376(1825).
69. Ren JF, Liu XA, Jiang F, et al. Unusual conservation of mitochondrial gene order in Crassostrea oysters: evidence for recent speciation in Asia. Bmc Evol Biol. 2010;10.
70. Wang JL, Li Q, Zhang JX, et al. High macro-collinearity between Crassostrea angulata and C. gigas genomes was revealed by comparative genetic mapping with transferable EST-SNP markers. Aquaculture. 2021;545.
71. Tan K, Liu HX, Ye T, et al. Growth, survival and lipid composition of Crassostrea gigas, C. angulata and their reciprocal hybrids cultured in southern China. Aquaculture. 2020;516.
72. Jiang GW, Li Q, Xu CX, et al. Reciprocal hybrids derived from Crassostrea gigas and C. angulata exhibit high heterosis in growth, survival and thermotolerance in northern China. Aquaculture. 2021;545.
73. Ghaffari H, Wang W, Li A, et al. Thermotolerance Divergence Revealed by the Physiological and Molecular Responses in Two Oyster Subspecies of Crassostrea gigas in China. Front Physiol. 2019;10.
74. Wang CG, Li A, Cong RH, et al. Cis- and Trans-variations of Stearoyl-CoA Desaturase Provide New Insights into the Mechanisms of Diverged Pattern of Phenotypic Plasticity for Temperature Adaptation in Two Congeneric Oyster Species. Mol Biol Evol. 2023;40(2).
75. Whitley D, Goldberg SP, Jordan WD. Heat shock proteins: A review of the molecular chaperones. J Vasc Surg. 1999;29(4):748-51.

Figure Legends

Figure 1 K-mer-based genome feature survey and sequencing reads partition.
a-c: GenomeScope analysis for C. angulata (AN), C. gigas (GI) and the offspring (CH1), respectively. d: The k-mer distribution at sizes of 25, 50, 75 and 100 bp for AN and GI short reads. e: The percentage of CH1 reads partition by AN and GI k-mers.

Figure 2 Genome assembling and assessment.
a-b: The heat map for Hi-C contact matrix of C. angulata and C. gigas, respectively. The colour scale in the heat map corresponds to the normalized reads counts representing the 3D proximity of pairs of contigs in the genome. A bright diagonal is the dominant visual feature indicating that most of the contigs were well placed. c: BUSCO evaluation and contig N50 of several published *Crassostrea* genomes. d: The schematic diagram of the 10 pairs of pseudo-chromosome sequences (A1,G1-A10,G10) of the two genomes.

Figure 3 The direct DNA sequence comparison between AN and GI genomes.
a: The dot plot for the direct comparison of AN and GI genomes. b: The synteny of conserved DNA blocks between the two genomes. c: The alignment identities of the 10 pairs of pseudo-chromosomes. d: A sketch map for the large indels in the alignments of conserved DNA segments.

Figure 4. Orthologous genes comparison between AN and GI genomes.
a: The number of orthologs of four subtypes in 15 genomes. b: The orthologous genes between *C. angulata* and *C. gigas*. Left: number distribution. Right: genomic position correlation. c: The identities of different gene regions. Top: Different regions/levels. Middle: up1k to up10k. Botom: dn1k to dn10k. d: The number of orthologous gene pairs which contained indels in different gene regions. Left: indel size >10 bp. Right: indel size >100 bp.

Figure 5 Phylogenetic and evolutionary analysis.
a: The phylogenetic tree and divergence time of *Crassostrea* species. b. The distribution of Ka, Ks and Ka/Ks. c. The expanded gene families. Left box: Expanded in C. angulata; Middle: Expanded in C. gigas; Right: Expanded in *C. angulata* and *C. gigas*.

Figure 6 Population data analysis.
a: Phylogenetic analysis of oysters from 5 populations. b. Population structure inference. c. The nucleotide diversity of *C. gigas* and *C. angulata*. d. Linkage disequilibrium decay analysis. e. The distribution of Fst and θπ ratio divergence.

Click here to access/download;Figure;f11.pdf

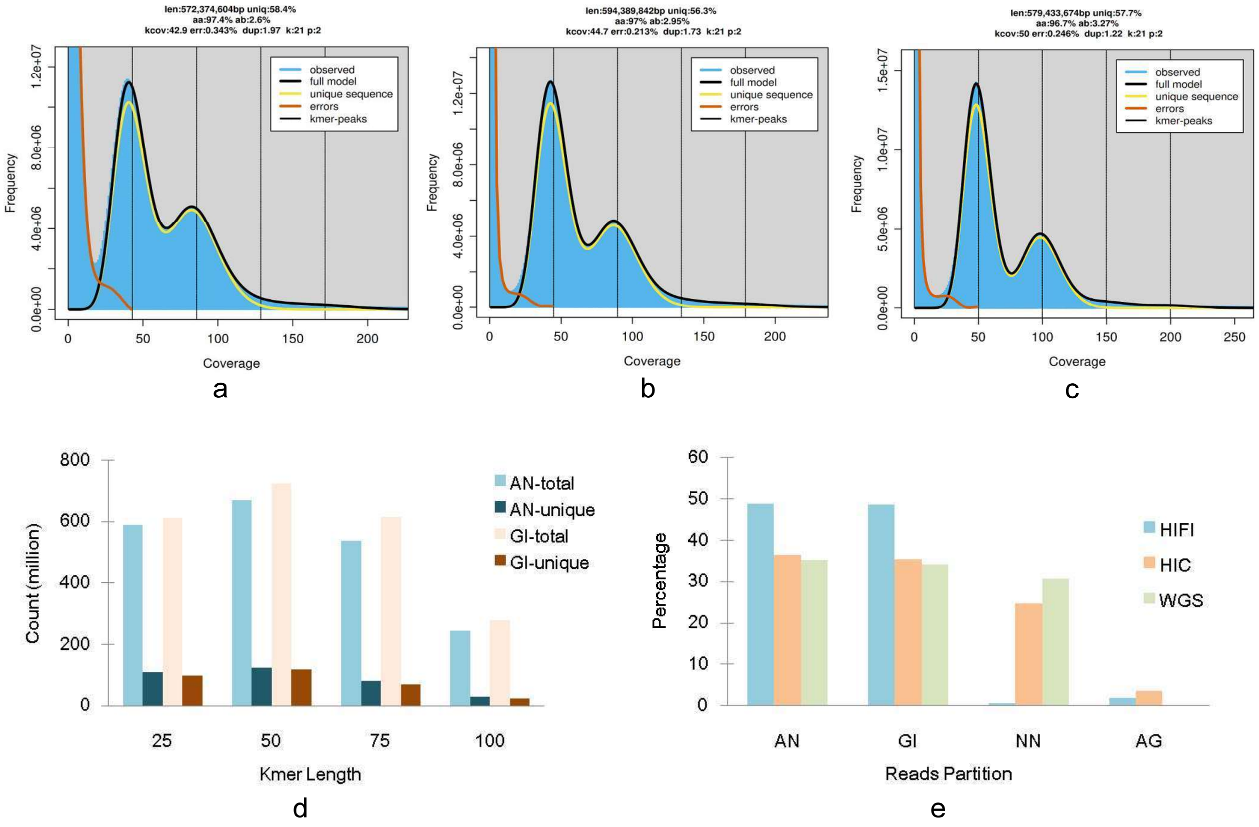

Figure 1

Figure2

Click here to access/download;Figure;f22.pdf

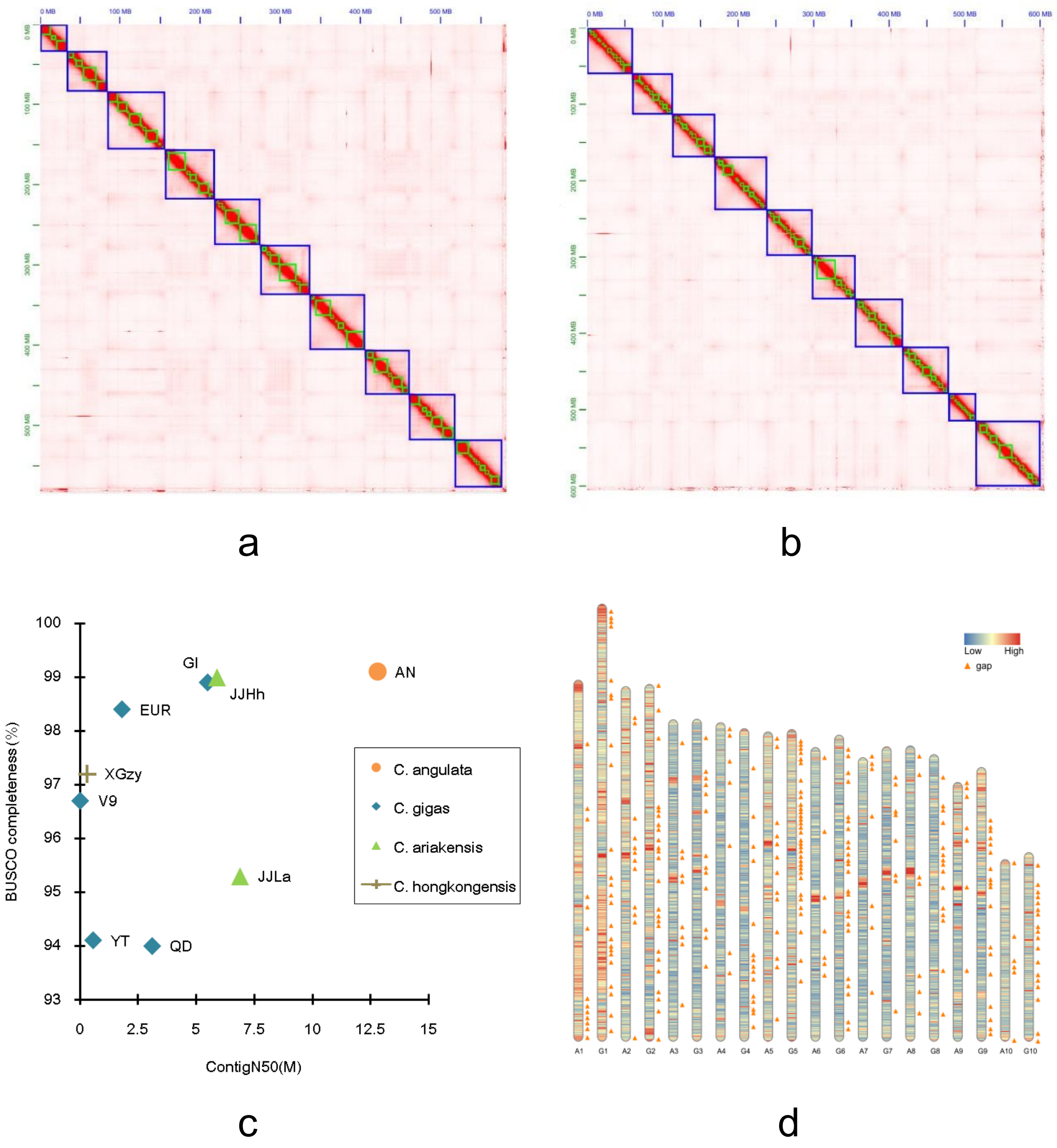

Figure3

Click here to access/download;Figure;f33.pdf

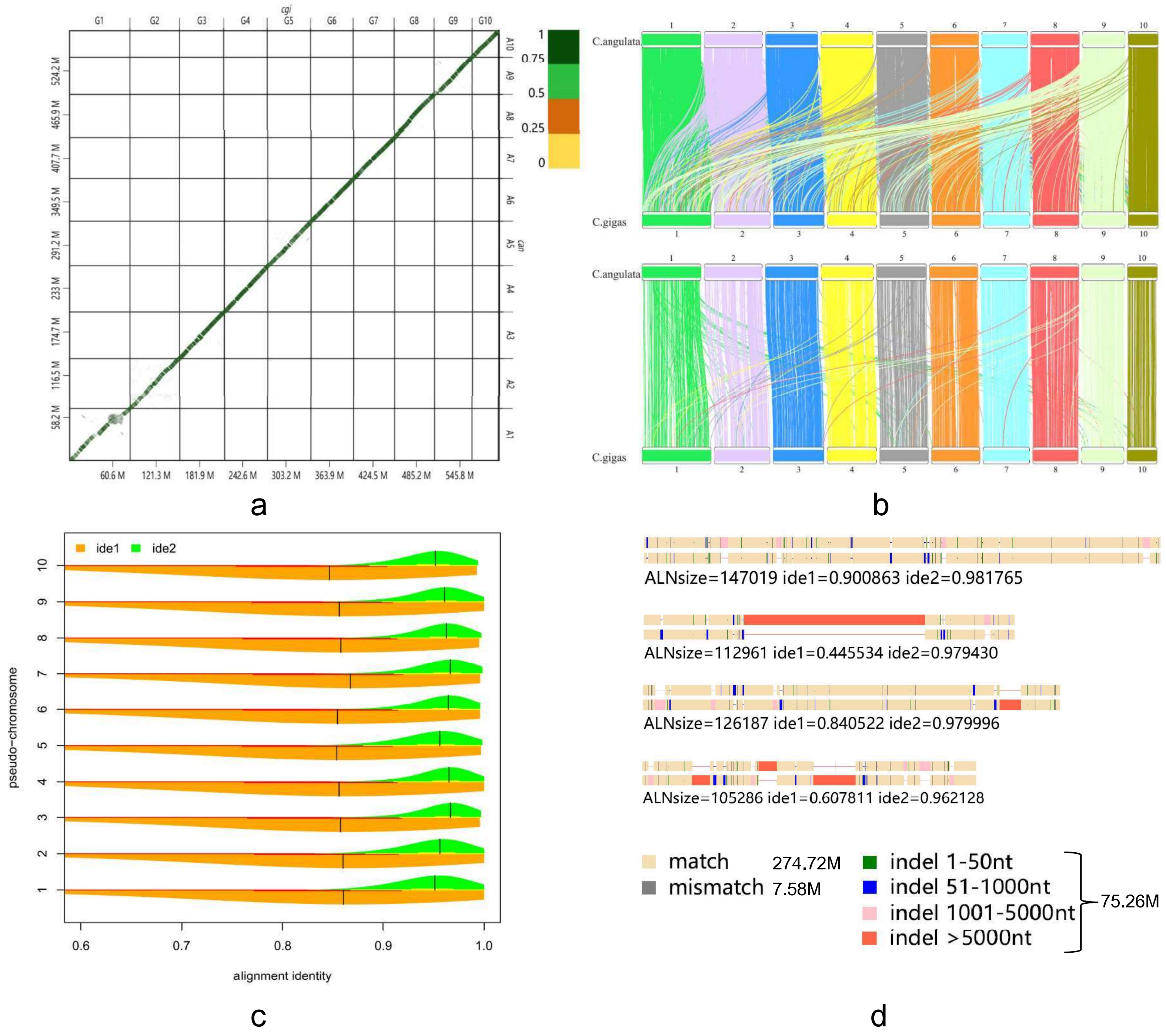

Figure 3

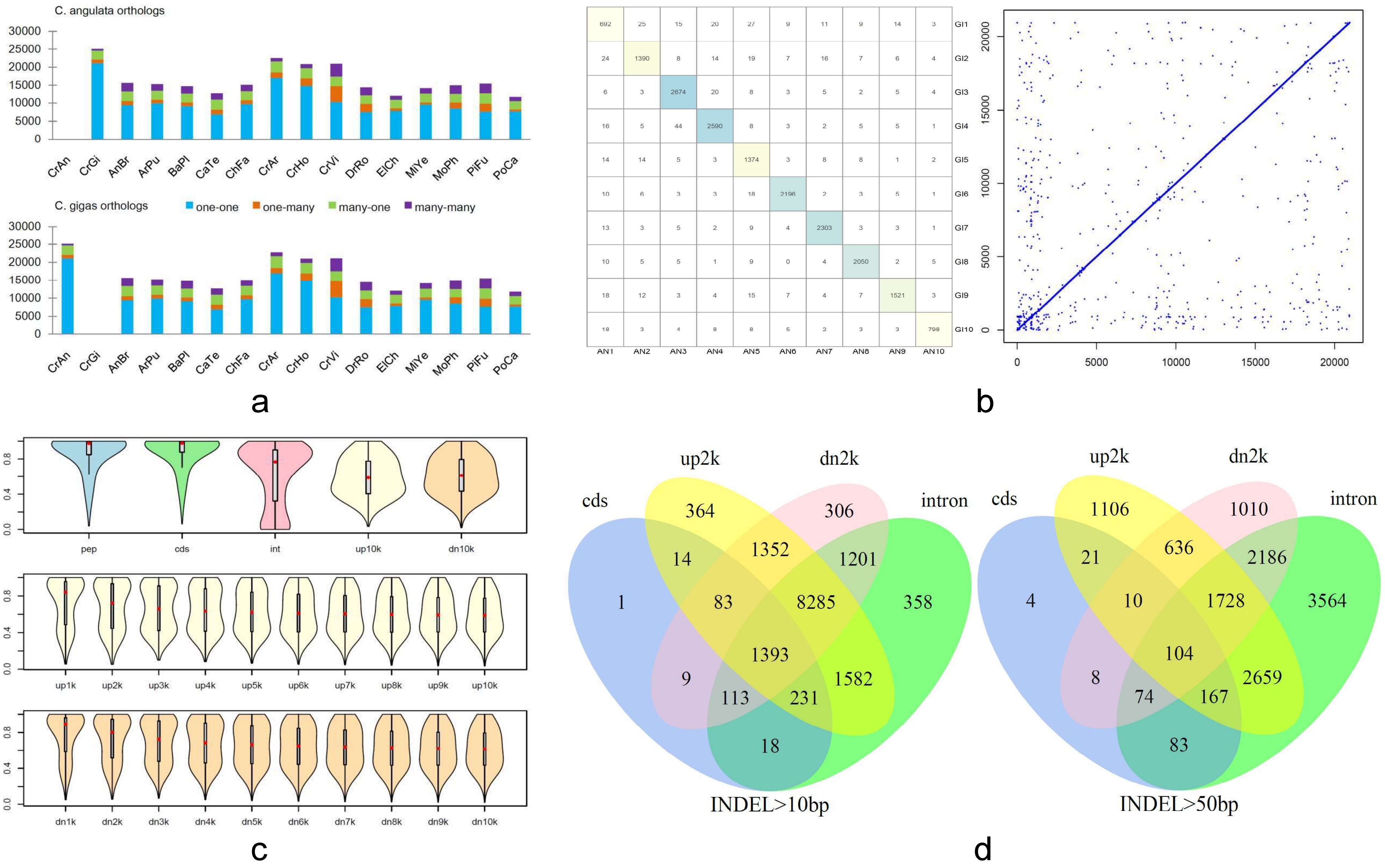
C. angulata orthologs
C. gigas orthologs
one-one
one-many
many-one
many-many
30000
25000
20000
15000
10000
5000
0
CrAn CrGi AnBr ArPu BaPl CaTe ChFa CrAr CrHo CrVi DrRo ElCh MiYe MoPh PiFu PoCa
a
692 25 15 20 27 9 11 9 14 3 GI1
24 1390 8 14 19 7 16 7 6 4 GI2
6 3 2674 20 8 3 5 2 5 4 GI3
16 5 44 2590 8 3 2 5 5 1 GI4
14 14 5 3 1374 3 8 8 1 2 GI5
10 6 3 3 18 2196 2 3 5 1 GI6
13 3 5 2 9 4 2303 3 3 1 GI7
10 5 5 1 9 0 4 2050 2 5 GI8
18 12 3 4 15 7 4 7 1521 3 GI9
18 3 4 8 8 5 2 3 3 798 GI10
AN1 AN2 AN3 AN4 AN5 AN6 AN7 AN8 AN9 AN10
0 5000 10000 15000 20000
b
pep cds int up10k dn10k
up1k up2k up3k up4k up5k up6k up7k up8k up9k up10k
dn1k dn2k dn3k dn4k dn5k dn6k dn7k dn8k dn9k dn10k
0.0 0.4 0.8
c
cds up2k dn2k intron
364 306 14 1352 1201 1 83 8285 358 1393 9 1582 113 231 18
INDEL>10bp
cds up2k dn2k intron
1106 1010 21 636 2186 4 10 1728 3564 104 8 2659 74 167 83
INDEL>50bp
d

Figure 4

Figure5

Click here to access/download;Figure;f55.pdf

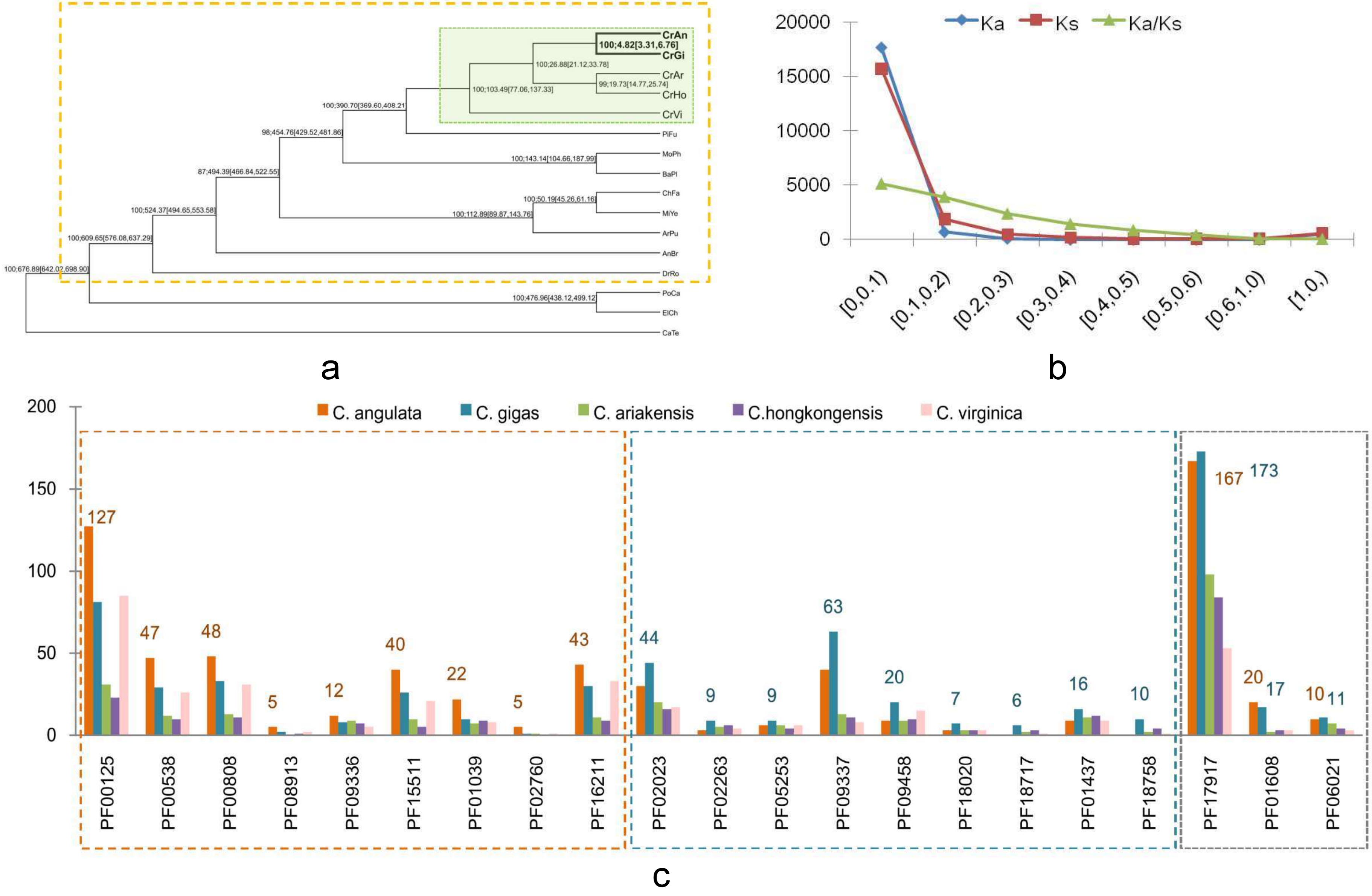

Figure 5

Click here to access/download;Figure;f66.pdf

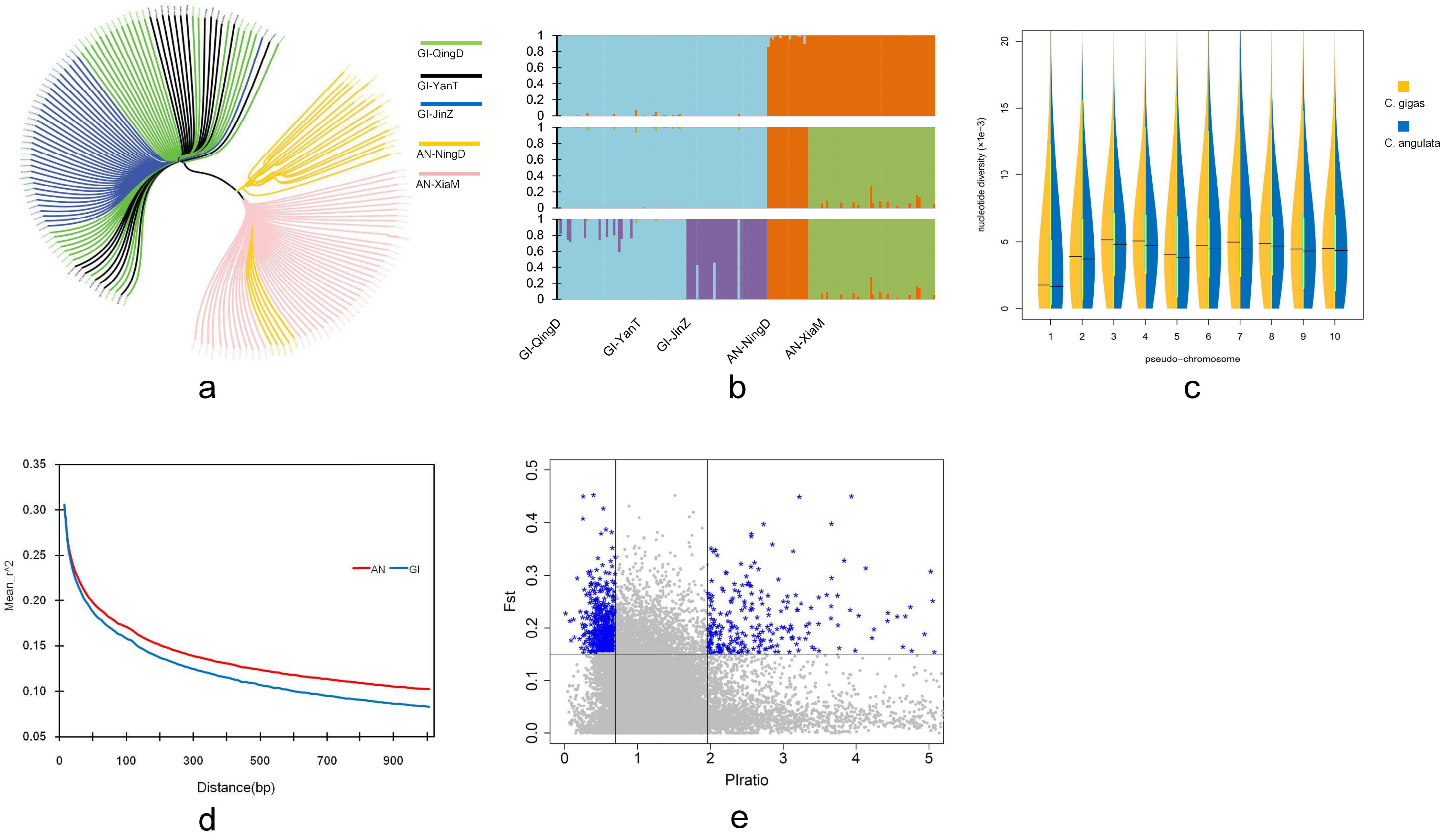

Figure 6

Supplementary file 1

Click here to access/download
Supplementary Material
SupplementaryFile1.txt

Supplementary file 2

Click here to access/download
Supplementary Material
SupplementaryFile2.txt

Table S1

Click here to access/download
Supplementary Material
Table_S1.xlsx

Table S2

Click here to access/download
Supplementary Material
Table_S2.xlsx

Table S3

Click here to access/download
Supplementary Material
Table_S3.xlsx

Table S4

Click here to access/download
Supplementary Material
Table_S4.xlsx

Table S5

Click here to access/download
Supplementary Material
Table_S5.xlsx

Table S6

Click here to access/download
Supplementary Material
Table_S6.xlsx

Table S7

Click here to access/download
Supplementary Material
Table_S7.xlsx
