# Supplementary material for: Construction and analysis of the chromosome-level haplotype-resolved genomes of two Crassostrea oyster congeners: Crassostrea angulata and Crassostrea gigas
Source: Gigascience. 2023 Oct 3;12:giad077. doi: 10.1093/gigascience/giad077 (PMC10546077; doi:10.1093/gigascience/giad077)
Supplement: giad077_Response_to_Reviewer_Comments_Original_Submission [file giad077_response_to_reviewer_comments_original_submission.pdf]

Author's Response To Reviewer Comments

Dear Hongfang Zhang,
We thank you and reviewers for the assessment of our manuscript "Construction and analysis of the chromosome-level haplotype-resolved genomes of two Crassostrea oyster congeners: Crassostrea angulata and C. gigas" (GIGA-D-23-00117).

We have revised the manuscript carefully according to the reviewers' comments and all the authors have approved the revised manuscript and agree with the submission. Please note that we have renewed the author's affiliation and the funding information in this revision.

We hope that the revised manuscript meets the requirement for publication in GigaScience and are looking forward to hearing from you.

Sincerely,
Haigang Qi

Reviewer #1: This manuscript presents two high quality and highly contiguous genome assemblies for congeners Crassostrea angulata and Crassostrea gigas using trio binning. The results here show the utility in applying new genome assembly strategies to overcome some persistent challenges in molluscan genome assemblies. I commend the authors for their work. This paper is written clearly and analyses are thoroughly explained. The methodological descriptions and information presented are sufficient, though I do think some further clarification on the phylogenetic dating analysis is required. Additionally, the figure legends, especially for the non-supplementary figures, are all missing key elements to understand the information presented.
Response:
Thanks for your valuable comments of our work. We have made a thorough revision for legends of all figures to provide sufficient information to understand them. Please see line 813-897.

Specific questions and comments:
Line 214-223. The PAML command and settings are not included here. The constraints for the dated analyses were taken from Time Tree but the references to support those calibrations are also not included.
Response:
As the settings for the running of PAML MCMCTree program contained many options, we provided the control file of MCMCTree as a new supplementary file 2 (line 221, the original supplementary file 2 was renamed as supplementary file 3), where the main parameters were [seed=-1 ndata=3 seqtype=0 usedata=1 clock=2 RootAge='' model=0 alpha=0 ncatG=5 cleandata=0 BDparas=1 1 0.1 kappa_gamma=6 2 alpha_gamma=1 1 rgene_gamma=2 20 1 sigma2_gamma=1 10 1 finetune=1: .1 .1 .1 .1 .1 .1 print=1 burnin=300000 sampfreq=10 nsample=100000].
We have added two references that directly provided the divergence time of C. gigas and C. virginica (line 222):
Towards a molecular phylogeny of Mollusks: Bivalves' early evolution as revealed by mitochondrial genes. Mol Phylogenet Evol. 2010;57(2):641-57.
Ren JF, Liu XA, Jiang F, et al. Unusual conservation of mitochondrial gene order in Crassostrea oysters: evidence for recent speciation in Asia. Bmc Evol Biol. 2010;10
We found that no references in TimeTree website directly provided the the divergence time of E. chlorotica and P. canaliculata and their divergence time was estimated by TimeTree database, and therefore we did not add references for the two species.

Line 815. This caption is a little unclear, more information should be added. The elements of each figure are not explained fully here, including the axes for each of the imaged. These should include full descriptions of what AN and GI represent.
Response:
We have updated the legend for Figure 1. Please see line 815-824. We have revised the figure caption to

"Genome feature survey and trio sequencing reads partition based on K-mer analysis" and more information including the axes and the key element descriptions were added.

Line 821. The legend should include which genomes are represented by the published genomes here. Not just the species they represent. For 2d, include Low-High refers to.
Response:
We have updated the legend for Figure 2. Please see line 826-843. For Fig2d, Low (blue) to High (red) gradual colors denote repeats content in 50-Kbp sliding windows. Orange triangles denote the gaps between contigs.

Line 830. Figure 3a, these are the dot plot comparisons of the full genome to genome alignments? Here again the axes should be defined and described in this figure caption. For 3b. what are the block sizes (minimum/maximum) shown here? For 3d. more information is needed to be able to fully understand this figure.
Response:
We have updated the legend for Figure 3. Please see line 845-862. Fig3a is the dot plot for the comparison of the 10 pairs of pseudo-chromosome sequences. Fig3b is the synteny of conserved DNA blocks between the two genomes. The two images were drawn from DNA blocks with size of ≥1,000 bp and ≥10,000 bp (max = 226,631 bp), respectively. For Fig3d, we have added the sequence alignments information.

Line 836. Again, this caption needs updating. The information presented is not sufficient. For 4c: "c: The identities of different gene regions. Top: Different regions/level Middle: up1k to up10k. Botom: dn1k to dn10k." I am unsure how to interpret this using the information presented here alone. Also, there is a spelling error here.
Response:
We have updated the legend for Figure 4. Please see line 864-877. Fig4c is the violin plot for sequence identities of different gene regions of orthologous genes. Top: the identities of protein sequences ('pep'), coding sequences ('cds'), introns ('int'), up-stream 10Kbp ('up10k') segments and down-stream 10Kbp ('dn10k') segments. Middle: the identities of 'up1k' to 'up10k' segments. Bottom: the identities of 'dn1k' to 'dn10k' segments. Typo 'Botom' has been corrected.

Line 844. Same as above, add information about each figure. E.g 5a, explain what node labels mean, what the brackets represent. For the 5a, including depictions of the confidence intervals using bars to scale could be included.
Response:
We have updated the legend for Figure 5. Please see line 879-885. We have made a minor revision for Fig5a, where the node label numbers denote the bootstrap value (percent), the estimated mean divergence time (Mya) and 95% confidence interval (in square brackets).

Line 850. Again here, expand on descriptions and add information about each element in the figure. See suggestions from previous comments.
Response:
We have updated the legend for Figure 6. Please see line 887-897.

Lastly, though I will leave this to the authors discretion, I noticed the paper both mollusc and mollusk are used interchangeably, I would suggest deciding on one spelling and using that throughout the paper.
Response:
We have changed 'mollusc' to 'mollusk' throughout the paper.

Reviewer #2: The authors have presented a high-quality assembly strategy and analyses to generate chromosome-level genomes for C. angulata and C. gigas. These genomes and the analyses will be of great value to the scientific community studying them. I only have a few comments:
Response:
Thanks for your valuable comments of the paper.

Line 36: I understand that the authors want to express that this is the first fully phased genome for marine invertebrates. But when you first read it it sounds like that the authors are considering this to be the first chromosome-level genome, which can be misleading. So I suggest rephrasing it to something like: "This is the first report of trio-binned fully-phased chromosome-level genomes in marine invertebrates".
Response:
We have revised the expression to "This is the first report of trio-binned fully-phased chromosome-level genomes in marine invertebrates." (line 37)

Line 67: rephrase "brought a substantial effect"
Response:
We have changed "brought a substantial effect on" to "brought convenience to". (line 67).

Line 77: two final points at the end of the line. Remove one.
Response:
We have removed one final point. (line 77)

Line 94-96: I'd rephrase the sentence about the Pacbio reads, if possible
Response:
We have changed "the PacBio high-fidelity (HiFi) reads" to "the PacBio high-fidelity (HiFi) sequencing method". (line 96)

Line 209-210: Am I correct to conclude that, given the orthofinder families with many-to-many, you took the genes with the same number of CDS to be the pairs you analysed on lines 224-227? If you could clarify this.
Response:
For the analysis of inferring the selective pressure (line 225-228), we used all the possible gene pairs in the many-to-many orthogroups. In the paper, for the sequence comparison of orthologous genes, we used all the possible gene pairs in the orthogroups. To clarify this, we changed the sentence "Genes with the same number of CDS were considered to have the same gene model structures" to "All the possible gene pairs in orthogroups were compared, and genes with the same number of CDS were considered to have the same gene model structures". (line 209)

Line 233:242. The analyses you did here are not a gene family analysis, rather, it is a protein domain-classification analysis. To do gene family evolutionary analysis, one would need to go from the orthofinder predicted orthogroups and run something like CAFE5. What the authors did was rather classify abundance of different protein domains in different gene families, as different gene families can share protein domains. The authors have done lots of other good analyses on this paper, so I suggest removing this (and then also lines 439-454) all together, or explicitly say they are analysing protein domain characterisation and abundance rather than gene families.
Response:
We agreed that it was a protein domain-classification based analysis rather than a proper gene family expansion and contraction analysis. We retained the analysis to maintain the integrity of the paper and according to your suggestion we called this part as "protein domain abundance analysis" and revised the words in corresponding positions. Please see line 234-239, 439-454 and 883-885.

Line 827-828: can authors explain in the figure legend and on the text what is the low and high in the pseudo chromosomes plot. Gene density?
Response:
We have made a thorough revision for legends of all figures to provide sufficient information to understand them. Please see line 813-897. In Fig2d, Low (blue) to High (red) gradual colors denote repeats content in 50-Kbp sliding windows. Orange triangles denote the gaps between contigs. (line 842-843)

Line 382: I don't understand Figure 3c very well. Can authors explaining what are ide1 and ide2?
387: could authors indicate on Figure 3d what pseudo chromosomes you are showing when showing the indels
Response:

Fig3c is the distribution of alignment identities of the conserved DNA blocks in the 10 pairs of pseudo-chromosomes. x-axis refers to sequence alignment identity. y-axis refers to the 10 pseudo-chromosome pairs. The black vertical lines in the bean plot denote the median values. 'ide1' denotes the alignment identity measured by gap-uncompressed method. 'ide2' denotes the alignment identity measured by gap-compressed method. In Fig3d, the four DNA alignments are from A3:13369498-13506783 vs G3:12755799-12900025, A5:4508362-4563006 vs G5:5295108-5404622, A6:20475358-20598634 vs G6:21302015-21412762, and A7:6838813-6932940 vs G7:7304889-7382157, respectively. (line 854-862)
