# Supplementary material for: Construction and analysis of the chromosome-level haplotype-resolved genomes of two Crassostrea oyster congeners: Crassostrea angulata and Crassostrea gigas
Source: Gigascience. 2023 Oct 3;12:giad077. doi: 10.1093/gigascience/giad077 (PMC10546077; doi:10.1093/gigascience/giad077)
Supplement: giad077_Reviewer_1_Report_Original_Submission — Vanessa Gonzalez, Ph.D. -- 6/23/2023 Reviewed [file giad077_reviewer_1_report_original_submission.pdf]

Reviewer Report

Title: Construction and analysis of the chromosome-level haplotype-resolved genomes of two Crassostrea oyster congeners: Crassostrea angulata and C. gigas

Version: Original Submission **Date:** 6/23/2023

Reviewer name: Vanessa Gonzalez, Ph.D.

Reviewer Comments to Author:

This manuscript presents two high quality and highly contiguous genome assemblies for congeners Crassostrea angulata and Crassostrea gigas using trio binning. The results here show the utility in applying new genome assembly strategies to overcome some persistent challenges in molluscan genome assemblies. I commend the authors for their work. This paper is written clearly and analyses are thoroughly explained. The methodological descriptions and information presented are sufficient, though I do think some further clarification on the phylogenetic dating analysis is required. Additionally, the figure legends, especially for the non-supplementary figures, are all missing key elements to understand the information presented.Specific questions and comments:Line 214-223. The PAML command and settings are not included here. The constraints for the dated analyses were taken from Time Tree but the references to support those calibrations are also not included.Line 815. This caption is a little unclear, more information should be added. The elements of each figure are not explained fully here, including the axes for each of the imaged. These should include full descriptions of what AN and GI represent.Line 821. The legend should include which genomes are represented by the published genomes here. Not just the species they represent. For 2d, include Low-High refers to.Line 830. Figure 3a, these are the dot plot comparisons of the full genome to genome alignments? Here again the axes should be defined and described in this figure caption. For 3b. what are the block sizes (minimum/maximum) shown here? For 3d. more information is needed to be able to fully understand this figure.Line 836. Again, this caption needs updating. The information presented is not sufficient. For 4c: "c: The identities of different gene regions. Top: Different regions/level Middle: up1k to up10k. Botom: dn1k to dn10k." I am unsure how to interpret this using the information presented here alone. Also, there is a spelling error here.Line 844. Same as above, add information about each figure. E.g 5a, explain what node labels mean, what the brackets represent. For the 5a, including depictions of the confidence intervals using bars to scale could be included.Line 850. Again here, expand on descriptions and add information about each element in the figure. See suggestions from previous comments.Lastly, though I will leave this to the authors discretion, I noticed the paper both mollusc and mollusk are used interchangeably, I would suggest deciding on one spelling and using that throughout the paper.

Level of Interest

Please indicate how interesting you found the manuscript: Choose an item.

Quality of Written English

Please indicate the quality of language in the manuscript: Choose an item.

Declaration of Competing Interests

Please complete a declaration of competing interests, considering the following questions:

- Have you in the past five years received reimbursements, fees, funding, or salary from an organisation that may in any way gain or lose financially from the publication of this manuscript, either now or in the future?
- Do you hold any stocks or shares in an organisation that may in any way gain or lose financially from the publication of this manuscript, either now or in the future?
- Do you hold or are you currently applying for any patents relating to the content of the manuscript?
- Have you received reimbursements, fees, funding, or salary from an organization that holds or has applied for patents relating to the content of the manuscript?
- Do you have any other financial competing interests?
- Do you have any non-financial competing interests in relation to this paper?

If you can answer no to all of the above, write 'I declare that I have no competing interests' below. If your reply is yes to any, please give details below.

I declare that I have no competing interests.

I agree to the open peer review policy of the journal. I understand that my name will be included on my report to the authors and, if the manuscript is accepted for publication, my named report including any attachments I upload will be posted on the website along with the authors' responses. I agree for my report to be made available under an Open Access Creative Commons CC-BY license (http://creativecommons.org/licenses/by/4.0/). I understand that any comments which I do not wish to be included in my named report can be included as confidential comments to the editors, which will not be published.

Choose an item.

To further support our reviewers, we have joined with Publons, where you can gain additional credit to further highlight your hard work (see: https://publons.com/journal/530/gigascience). On publication of this paper, your review will be automatically added to Publons, you can then choose whether or not to claim your Publons credit. I understand this statement.

Yes Choose an item.
