# Supplementary material for: Construction and analysis of the chromosome-level haplotype-resolved genomes of two Crassostrea oyster congeners: Crassostrea angulata and Crassostrea gigas
Source: Gigascience. 2023 Oct 3;12:giad077. doi: 10.1093/gigascience/giad077 (PMC10546077; doi:10.1093/gigascience/giad077)
Supplement: giad077_Reviewer_2_Report_Original_Submission — Marcela Uliano-Silva -- 7/12/2023 Reviewed [file giad077_reviewer_2_report_original_submission.pdf]

Reviewer Report

Title: Construction and analysis of the chromosome-level haplotype-resolved genomes of two Crassostrea oyster congeners: Crassostrea angulata and C. gigas

Version: Original Submission **Date:** 7/12/2023

Reviewer name: Marcela Uliano-Silva

Reviewer Comments to Author:

The authors have presented a high-quality assembly strategy and analyses to generate chromosome-level genomes for C. angulata and C. gigas. These genomas and the analyses will be of great value to the scientific community studying them. I only have a few comments:Line 36: I understand that the authors want to express that this is the first fully phased genome for marine invertebrates. But when you first read it it sounds like that the authors are considering this to be the first chromosome-level genome, which can be misleading. So I suggest rephrasing it to something like: "This is the first report of trio-binned fully-phased chromosome-level genomes in marine invertebrates".Line 67: rephrase "brought a substantial effect"Line 77: two final points at the end of the line. Remove one.Line 94-96: I'd rephrase the sentence about the Pacbio reads, if possibleLine 209-210: Am I correct to conclude that, given the orthofinder families with many-to-many, you took the genes with the same number of CDS to be the pairs you analysed on lines 224-227? If you could clarify this.Line 233:242. The analyses you did here are not a gene family analysis, rather, it is a protein domain-classification analysis. To do gene family evolutionary analysis, one would need to go from the orthofinder predicted orthogroups and run something like CAFE5. What the authors did was rather classify abundance of different protein domains in different gene families, as different gene families can share protein domains. The authors have done lots of other good analyses on this paper, so I suggest removing this (and then also lines 439-454) all together, or explicitly say they are analysing protein domain characterisation and abundance rather than gene families.Line 827-828: can authors explain in the figure legend and on the text what is the low and high in the pseudo chromosomes plot. Gene density?Line 382: I don't understand Figure 3c very well. Can authors explaining what are ide1 and ide2?387: could authors indicate on Figure 3d what pseudo chromosomes you are showing when showing the indelsCongratulations on the work once again.Best,Marcela.

Level of Interest

Please indicate how interesting you found the manuscript: Choose an item.

Quality of Written English

Please indicate the quality of language in the manuscript: Choose an item.

Declaration of Competing Interests

Please complete a declaration of competing interests, considering the following questions:

- Have you in the past five years received reimbursements, fees, funding, or salary from an organisation that may in any way gain or lose financially from the publication of this manuscript, either now or in the future?
- Do you hold any stocks or shares in an organisation that may in any way gain or lose financially from the publication of this manuscript, either now or in the future?
- Do you hold or are you currently applying for any patents relating to the content of the manuscript?
- Have you received reimbursements, fees, funding, or salary from an organization that holds or has applied for patents relating to the content of the manuscript?
- Do you have any other financial competing interests?
- Do you have any non-financial competing interests in relation to this paper?

If you can answer no to all of the above, write 'I declare that I have no competing interests' below. If your reply is yes to any, please give details below.

I declare that I have no competing interests

I agree to the open peer review policy of the journal. I understand that my name will be included on my report to the authors and, if the manuscript is accepted for publication, my named report including any attachments I upload will be posted on the website along with the authors' responses. I agree for my report to be made available under an Open Access Creative Commons CC-BY license (http://creativecommons.org/licenses/by/4.0/). I understand that any comments which I do not wish to be included in my named report can be included as confidential comments to the editors, which will not be published.

Choose an item.

To further support our reviewers, we have joined with Publons, where you can gain additional credit to further highlight your hard work (see: https://publons.com/journal/530/gigascience). On publication of this paper, your review will be automatically added to Publons, you can then choose whether or not to claim your Publons credit. I understand this statement.

Yes Choose an item.
